# Synthesis of “Nereid,” a new phenol‐free detergent to replace Triton X‐100 in virus inactivation

**DOI:** 10.1002/jmv.26708

**Published:** 2020-12-17

**Authors:** Jean‐Baptiste Farcet, Johanna Kindermann, Michael Karbiener, Richard Scheinecker, Otto Kostner, Thomas R. Kreil

**Affiliations:** ^1^ Pharmaceutical Sciences, Baxalta Innovations GmbH, now part of the Takeda Group of Companies Vienna Austria; ^2^ Global Pathogen Safety, Baxter AG, now part of the Takeda Group of Companies Vienaa Austria

**Keywords:** detergent, organic synthesis, structure–activity relationship, Triton X‐100, virus inactivation

## Abstract

In the 1980s, virus inactivation steps were implemented into the manufacturing of biopharmaceuticals in response to earlier unforeseen virus transmissions. The most effective inactivation process for lipid‐enveloped viruses is the treatment by a combination of detergents, often including Triton X‐100 (TX‐100). Based on recent environmental concerns, the use of TX‐100 in Europe will be ultimately banned, which forces the pharmaceutical industry, among others, to switch to an environmentally friendly alternative detergent with fully equivalent virus inactivation performance such as TX‐100. In this study, a structure–activity relationship study was conducted that ultimately led to the synthesis of several new detergents. One of them, named “Nereid,” displayed inactivation activity fully equivalent to TX‐100. The synthesis of this replacement candidate has been optimized to allow for the production of several kg of detergent at lab scale, to enable the required feasibility and comparison virus inactivation studies needed to support a potential future transition. The 3‐step, chromatography‐free synthesis process described herein uses inexpensive starting materials, has a robust and simple work‐up, and allows production in a standard organic laboratory to deliver batches of several hundred grams with >99% purity.

## Graphical abstract

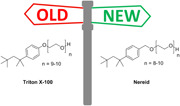

## 1 INTRODUCTION

Following the transmission of viruses through plasma derivatives in the early 1980s, the solvent/detergent (S/D) treatment was widely implemented to inactivate lipid‐enveloped viruses.^1^, ^2^ To minimize the risk of transmitting infectious lipid‐enveloped viruses like the hepatitis B or C viruses (HBV, HCV), Human immunodeficiency virus (HIV), and also emerging and unknown viruses, S/D treatment is still recognized as the most effective and robust method.^3^ Frequently, this mild chemical treatment contains a mixture of the non‐ionic detergent TX‐100 (**I**), tri‐n‐butyl‐phosphate (TNBP), and often also Polysorbate 80 (PS80).

More recently it has been realized that TX‐100 (**I**), once released into the environment, generates environmentally unfriendly metabolites. In particular, the phenol moiety of TX‐100 (4‐(1,1,3,3)‐tetramethylbutylphenol) is resistant to biodegradation and as such toxic to the environment, for example, it may impair the reproductive cycles of aquatic organisms.^4^ These observations have resulted in restrictions on its use through the European Chemicals Agency.^5^, ^6^ The pharmaceutical industry as well as other large consumers of TX‐100 (**I**)^7^, ^8^ now face the challenge to find an alternative detergent to eventually replace TX‐100 (**I**), and indeed, some alternative detergents have recently been suggested.^9^, ^10^, ^11^

In order to not to adversely affect pharmaceutical bioprocesses, the best solution would be to replace TX‐100 (**I**) with a detergent of analogous activity and behavior, thus minimizing any impact on the virus inactivation capacity, physical behavior, and removability during subsequent manufacturing steps, to ultimately support a smooth transition away from the phenol‐containing detergent TX‐100 (**I**). As detergents with a close structural similarity to TX‐100 (**I**) were not available, an attempt was made to develop non‐phenolic TX‐100‐like detergents and to evaluate their ability to inactivate lipid‐enveloped viruses.

## 2 MATERIALS AND METHODS

### 2.1 Process intermediates

As a model matrix for plasma‐derived products, Fraction II was used, that is, the process intermediate obtained during immunoglobulin production at the stage before virus inactivation/reduction steps.^22^ The starting material had a pH of 5.2. Immediately before virus inactivation runs, the material was filtered through a 0.2 µm filter, and the protein concentration was adjusted to 28.9 AU_280‐320_/cm with 30 mM NaCl. As a model matrix for recombinant protein production, human albumin (25%, Baxter AG) was diluted to 0.6 mg/ml using a buffer that contained 396 mM NaCl, 20 mM MES acid monohydrate, 10 mM CaCl_2_ dihydrate, and 0.099% (v/v) PS80. This matrix is representative for an intermediate of the production process of recombinant factor VIII (ADVATE; Baxter AG). The starting material had a pH of 6.4 and was filtered through a 0.2 µm filter immediately before virus inactivation runs were performed.

### 2.2 Cell culture, virus propagation, and titration

Vero cells (European Collection of Authenticated Cell Cultures; 84113001) were propagated in an in‐house manufactured basal medium that was supplemented with 5% fetal calf serum (FCS; PAN‐Biotech), 2 mM l‐glutamine, 1 mM sodium pyruvate, 0.75% sodium bicarbonate, 1x non‐essential amino acids, and 100 µg/ml gentamicin sulfate (all GibcoTM, Thermo Fisher Scientific). Mus dunni cells (American Type Culture Collection [ATCC]; CRL‐2017) and PG4 cells (ATCC; CRL‐2032) were propagated in Mc Coy's 5A Medium that was supplemented with 10% FCS, 2 mM l‐glutamine, and 100 µg/ml gentamicin sulfate.

Pseudorabies virus (PRV; strain Kaplan) was obtained from Eberhard Karls University, Tübingen, Germany. The virus was propagated on Vero cells, which were also used as an indicator cell line for virus titrations. Xenotropic Murine Leukemia Virus (X‐MuLV; strain pNFS Th‐1) was obtained from the ATCC (VR‐1447). The virus was propagated on Mus dunni cells and titrated on PG4 cells.

The choice of viruses reflects requirements as defined in the relevant guidelines^23^, ^24^: PRV was included as a model for the family of Herpesviridae, while X‐MuLV served as a model for retroviruses, which are of concern as retroviral‐like particles have been reported in rodent cell lines utilized for recombinant protein production. Virus stocks were produced from infected susceptible cell lines essentially as earlier described.^25^, ^26^ For titration of virus infectivity, median tissue culture infectious dose (TCID_50_) assays were employed using eightfold replicates of serial half‐log sample dilutions of virus‐containing samples that were incubated on the respective indicator cell lines. After incubation, cytopathic effects were evaluated by microscopic visual inspection. TCID_50_ titers were calculated according to the Poisson distribution and expressed as log_10_[TCID_50_/ml]. Virus reduction factors were calculated in accordance with the EU Committee for Proprietary Medicinal Products guidance.^27^

### 2.3 Virus inactivation by three‐component S/D treatment

Each combination of S/D mix, virus, and matrix was investigated in duplicate runs using 30–50 ml of filtered process material per run. Throughout the entire experimental runs, the process material was kept at 17 ± 1°C (plasma‐derived model matrix) or 1 ± 1°C (recombinant model matrix) and continuously mixed by a magnetic stirrer. Spiking with the respective virus stock solution was performed at a ratio of 1:31 (v/v). Subsequently, two samples termed spike control (SC) and hold control (HC) were drawn. SC was titrated immediately while HC was incubated in the same cooling unit as the SD‐treated process material and titrated at the end of the respective run. For the vast majority of experiments, the difference in virus titer between SC and HC pairs was ≤0.5 log_10_[TCID_50_/ml], indicating that neither matrix constituents nor the chosen physicochemical parameters led to virus inactivation. As for manufacturing, the final target concentrations of PS80, TNBP, and TX‐100 are 0.3%, 0.3%, and 1% (w/w), respectively. Taking the ratio of SD components into account, different S/D mixes were prepared by combining PS80 (Merck; 817061) and TNBP (Merck; 100002) with either TX‐100 (Merck; 108643) or the potential surrogate compounds. Among these substances, TX‐100 reduced (Sigma Aldrich; X100RS), Ecosurf EH‐9, and Tergitol TNM‐100X (both Dow Chemicals) were obtained commercially, while the other detergents were synthesized in‐house (see ESI). The spiked process material was weighed to calculate the amount of S/D mix to be added to reach 5% (plasma‐derived model matrix) or 10% (recombinant model matrix) of the concentration as specified for manufacturing. These considerably reduced final concentrations enable to demonstrate virus inactivation kinetics, a regulatory requirement,^27^ as opposed to virtually immediate virus inactivation at manufacturing concentrations. The detergent mix was added with a Hamilton syringe, and the actual amount of detergent added was determined by back‐weighing the syringe. Samples for TCID_50_ virus titration were drawn at 1–2, 10 ± 1, 30 ± 1, and 59 ± 1 min after detergent addition.

For all used process materials, the cytotoxicity of the respective S/D mix, as well as any possible matrix effects on cell lines used for virus detection were tested and taken into consideration for the calculation of reduction factors.

## 3 RESULTS AND DISCUSSION

### 3.1 Looking for a bioisostere

Bioisosteres are surrogate compounds designed to exhibit similar physical properties with broadly similar biological activity to another chemical compound. The purpose of exchanging one bioisostere for another can be multiple such as improved potency, enhanced selectivity, altered physical properties, reduced or redirected metabolism, acquisition of novel intellectual property or circumvention of undesired metabolites.^12^ The present investigation was based on the need to eliminate or modify the phenol toxicophore present in TX‐100 (**I**) while retaining its full inactivation activity against viruses as well as its well‐established process compatibility.

Bioisosteres possess near‐equal molecular shapes and volumes and have approximately the same electron distribution.^13^ As a previous study revealed that none of the tested, commercially available detergents fulfilled these prerequisites,^11^ a library of new detergents for virus inactivation was established, to screen for novel TX‐100 (**I**) replacement compounds.

### 3.2 Structure–activity relationship (Sar)

It is appealing to understand which structural elements of TX‐100 (**I**) are responsible for its outstanding virus inactivation activity,^14^ as this subject has not been addressed in the public domain since its discovery.^1^ Ultimately, understanding the role of each functional group in the structure might be crucial to find the most suitable replacement detergent.

In addition to its polyethylene glycol chain, TX‐100 (**I**) features structural elements such as the 1,1,3,3‐tetramethylbutyl alkyl chain, the aromatic ring, and the linkage to the polyethylene glycol chain. All those elements can be chemically modified one by one by designed chemical synthesis, to reveal their contribution to the activity in subsequent virus inactivation studies.

### 3.3 Sar#1: Removing the aromaticity

**Figure 1:**
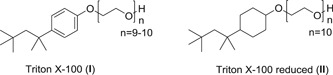
Structures of Triton X‐100 (TX‐100) and TX‐100 reduced

By removing the aromaticity from the structure of TX‐100 (**I**), we obtained the structure of TX‐100 reduced (**II**), a commercially available detergent (Figure 1).

**Figure 2:**
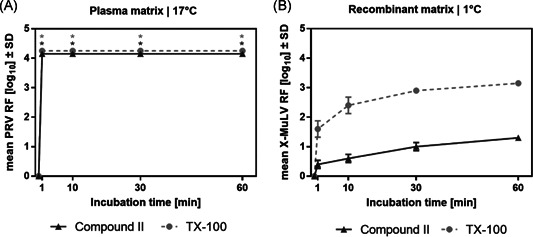
Virus inactivation by S/D mixes containing TX‐100 versus compound II (TX‐100 reduced). A, Plasma‐derived model matrix was spiked with PRV before the addition of S/D mix consisting of PS80, TNBP, and TX‐100 or compound II. Duplicate runs were performed at 17°C at a final S/D mix concentration of 5% as specified for manufacturing. B, Recombinant model matrix was spiked with Xenotropic murine leukemia virus (X‐MuLV) before the addition of S/D mix consisting of PS80, TNBP, and TX‐100 or compound II. Duplicate runs were performed at 1°C at a final S/D mix concentration of 10% as specified for manufacturing. A, B, Samples were drawn after 1, 10, 30, and 60 min; viral loads of these samples were compared to a sample drawn before S/D mix addition to calculate the respective RF. Asterisks indicate viral inactivation below the detection limit. *SD* only shown if larger than the height of symbols). PRV, pseudorabies virus; RF, reduction factor; S/D, solvent/detergent; *SD*, standard deviation; TNBP, tri‐n‐butyl‐phosphate; TX‐100, Triton X‐100

The compound shows excellent virus inactivation when S/D treatment was performed at standard process temperature and in a matrix representative for a plasma‐derived product. As for recombinant proteins, lower temperatures are preferable during the downstream processes, and therefore, it is also important to investigate the effectivity of S/D treatment under these conditions. However, TX‐100 reduced (**II**) failed to effectively inactivate a model retrovirus in a recombinant model matrix at such cold temperature (Figure 2).

It is tempting to speculate that TX‐100 reduced (**II**) has a diminished penetrating activity compared to TX‐100 (**I**), which is both efficient at low and elevated temperature in plasmatic and recombinant matrices. A possible cause for the more moderate inactivation at low temperature might be the decrease of the virus membrane fluidity and its porosity as described in the fluid mosaic model.^15^

### 3.4 Sar#2: Removing the entire ring structure

In a further SAR step, the whole ring was removed to yield model compound **III**, having an almost identically substituted alkyl chain as TX‐100 (**I**) directly linked to the hydrophilic polyethylene glycol chain.

**Scheme 1:**
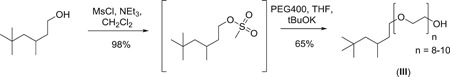
Synthesis route of detergent **III**

To access compound **III**, the corresponding highly substituted alcohol was mesylated followed by nucleophilic attack of a monoprotonated PEG polymer, which delivered the candidate **III** in a satisfactory 64% overall yield after a chromatographic purification in multigram scale (Scheme 1).

**Figure 3:**
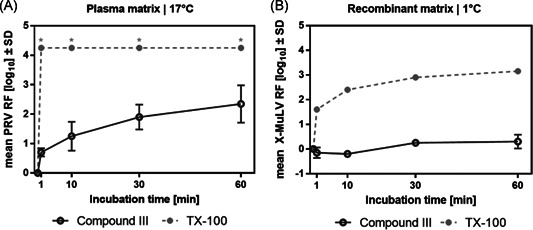
Virus inactivation by S/D mixes containing novel compound III. Matrices were spiked with the respective virus before the addition of S/D mix (PS80, TNBP, compound III). Samples were drawn 1, 10, 30, and 60 min after S/D mix addition; viral loads of these samples were compared to a sample drawn before S/D mix addition to calculate the respective RF. A, Duplicate runs were performed at 17°C using a plasma‐derived model matrix and PRV; the final S/D mix concentrations were 5% of manufacturing. Asterisks indicate viral inactivation below detection limit. B, Duplicate runs were performed at 1°C using a recombinant model matrix and Xenotropic murine leukemia virus (X‐MuLV); the final S/D mix concentrations were 10% of manufacturing. *SD* only shown if larger than the height of symbols). PRV, pseudorabies virus; RF, reduction factor; S/D, solvent/detergent; *SD*, standard deviation; TNBP, tri‐n‐butyl‐phosphate; TX‐100, Triton X‐100

Interestingly, the inactivation properties of compound **III** were rather limited at warm temperature and negligible at cold conditions (Figure 3). These results corresponded to analyses of the recently proposed TX‐100 (**I**) surrogates Ecosurf EH‐9 and Tergitol TNM‐100X (both containing branched relatively short alkyl chains similar to **III** as lipophilic part) which equally failed to show significant inactivation, especially at a cold temperature (data not shown).

A hypothesis for the low activity of structures without the aromatic ring might be that the planar geometry of the aromatic ring enables to create a denser interaction with the virus membrane, by π–π stacking and/or hydrophobic interaction with membrane proteins, for instance, thereby favoring the formation of mixed micelles and disruption of the lipid envelope.

### 3.5 Sar#3: Adding π‐electrons to the system

**Figure 4:**
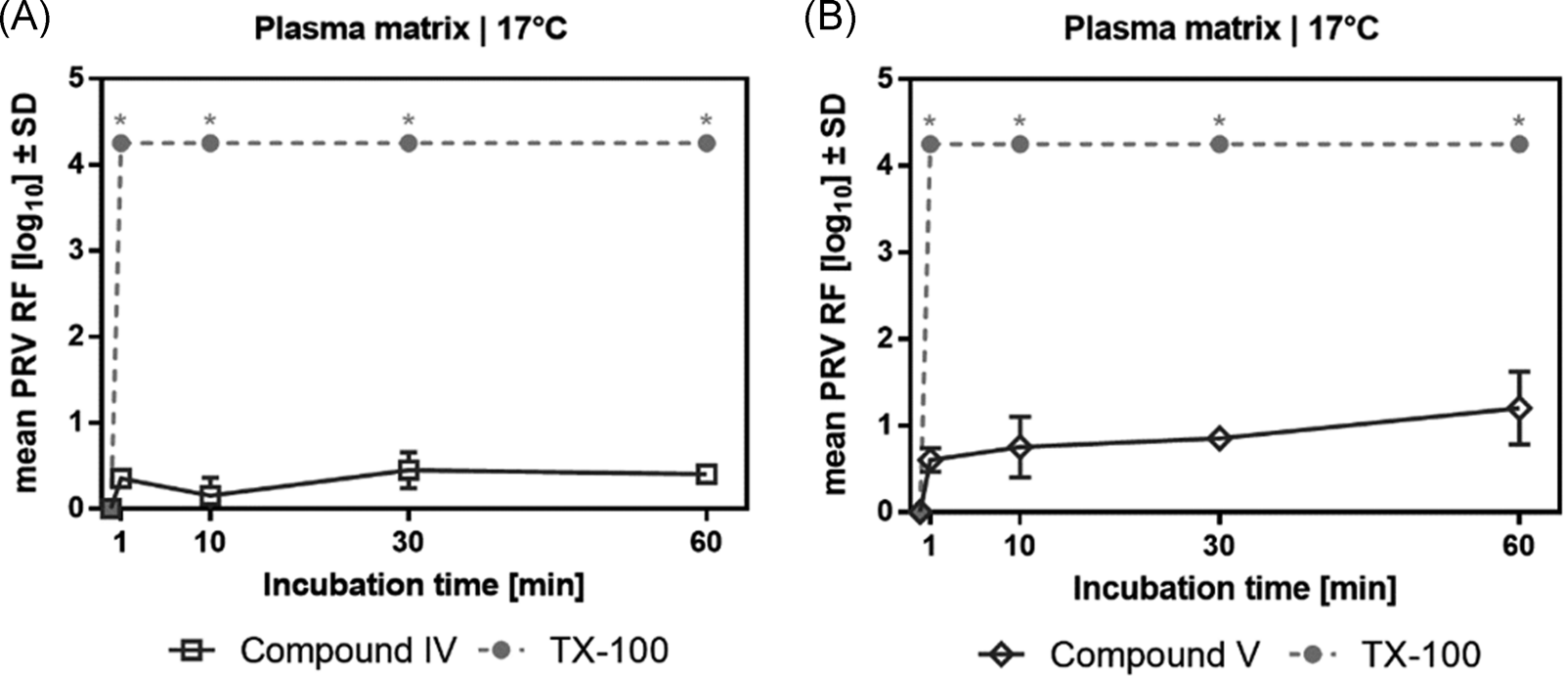
Virus inactivation by S/D mixes containing novel compounds IV and V. Duplicate runs were performed at 17°C using a plasma‐derived model matrix and PRV. PS80 and TNBP were combined with (A) compound IV or (B) compound V and added to the virus‐spiked matrix to reach a final concentration of 5% as specified for manufacturing. A, B, Samples were drawn 1, 10, 30, and 60 min after S/D mix addition; viral loads of these samples were compared to a sample drawn before S/D mix addition to calculate the respective reduction factor (RF). Asterisks indicate viral inactivation below detection limit. *SD* only shown if larger than the height of symbols). PRV, pseudorabies virus; RF, reduction factor; S/D, solvent/detergent; *SD*, standard deviation; TNBP, tri‐n‐butyl‐phosphate; TX‐100, Triton X‐100

**Scheme 2:**
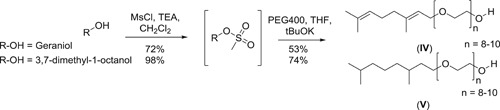
Synthesis route of detergents **IV** and **V**

Next, an electron richer detergent **IV** containing two double bonds, based on the Geraniol structure as well as its fully hydrogenated derivative **V** were synthesized in a similar manner in multigram scale and in acceptable overall yield (38% and 73% respectively, Scheme 2). For both compounds, virus inactivation was unsatisfactory already at standard temperature (Figure 4), hence an analysis as low temperature was omitted. Overall, these results did not confirm that extra π electrons in the detergent structure assist pathogen reduction.

### 3.6 Sar#4: Restoring the aromaticity but changing linkage to the PEG chain

At this point of the investigation, we had elaborated the crucial presence of the aromatic ring in the detergent structure to reach maximum virus inactivation and more information was required to evaluate the role of other elements, such as the *tert*‐octyl chain as well as the phenol linkage to the polyethylene glycol chain. To gain more insight, compounds **VI** and **VII** were synthesized, in which the aromatic planar ring was restored. Although **VI** has the same structure as TX‐100 (**I**) with the sole exception of having an extra methylene group inserted between the aromatic ring and the polyethylene glycol chain, **VII** features, in addition to the latter change, a shortened alky chain (tert‐butyl instead of tert‐octyl para‐substituted to the aromatic ring).

**Scheme 3:**
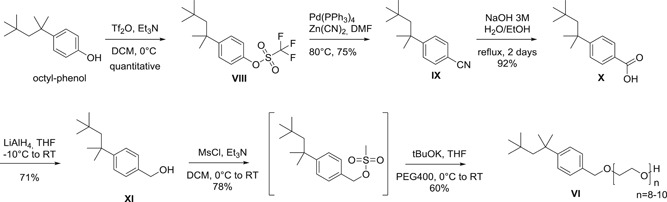
Six‐step synthesis of detergent **VI**

To synthesize compound **VI**, a 6‐step synthesis was envisaged taking advantage of the already established octyl alky chain in the para position of the aromatic ring in the starting material octyl‐phenol. First, the corresponding triflate intermediate **VIII** was obtained quantitatively using triflate anhydride. Next, a palladium catalyzed reaction in combination with a stoichiometric amount of zinc(II) cyanide delivered, after a chromatographic step, the para‐substituted benzonitrile **IX**. Hydrolysis followed by reduction of the carboxylic acid **X** yielded the benzyl alcohol **XI** as a solid. The last 2 steps, following similar protocols as described above, finalized the synthesis of over 120 g of **VI** with an overall yield of almost 23% over 6 steps (Scheme 3).

**Scheme 4:**
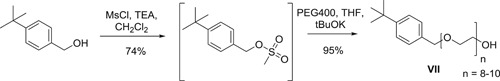
Synthesis route of detergent **VII**

Surfactant **VII** could be obtained in good overall yield (70%) from the commercially available *tert*‐butyl benzyl alcohol following standard mesylation/nucleophilic substitution, described previously in gram scale (Scheme 4).

**Figure 5:**
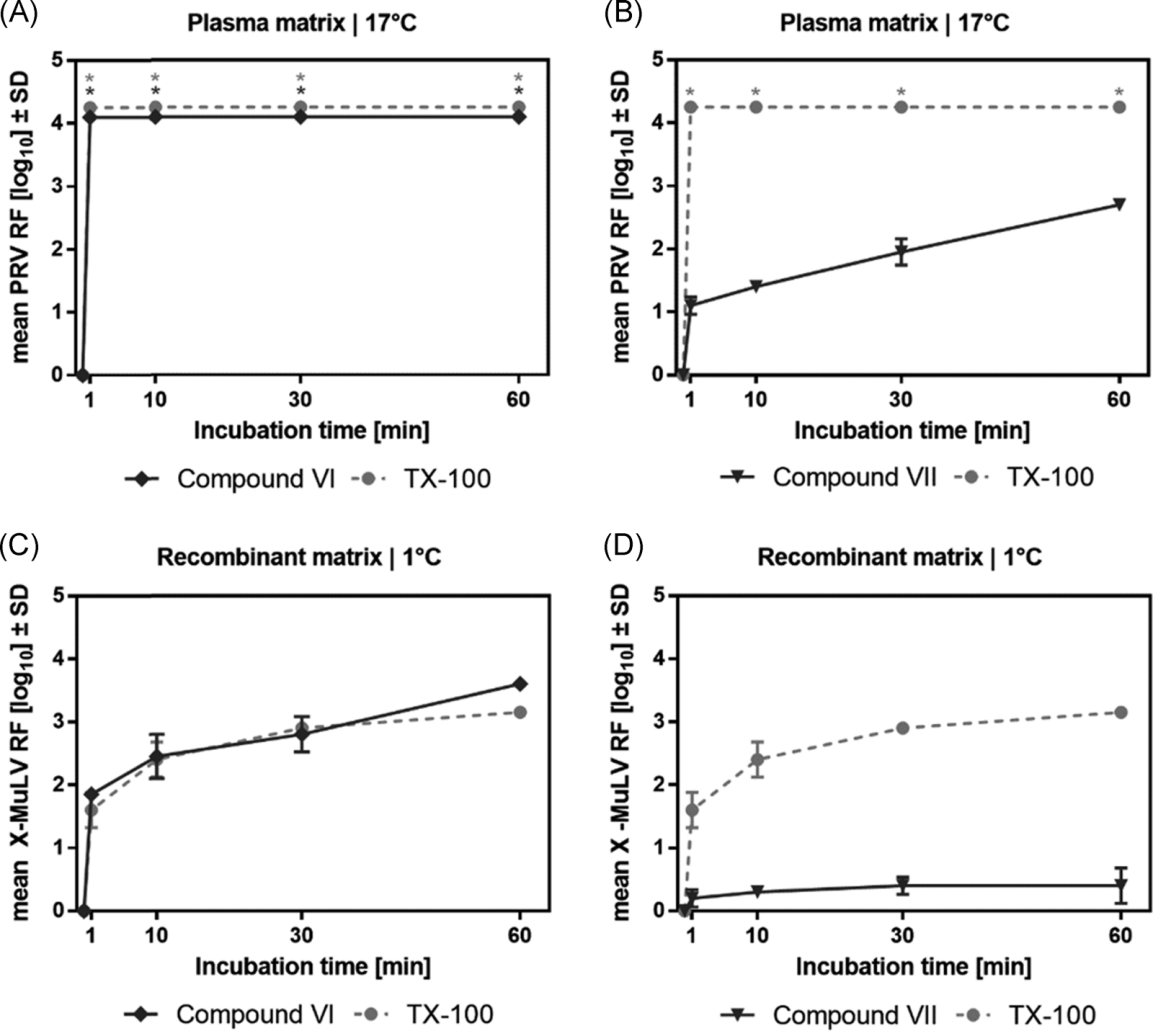
Virus inactivation by S/D mixes containing novel compounds VI and VII. Matrices were spiked with the respective virus before addition of S/D mix (PS80, TNBP, compound VI/compound VII). Samples were drawn 1, 10, 30, and 60 min after S/D mix addition; viral loads of these samples were compared to a sample drawn before S/D mix addition to calculate the respective RF. A, B, Duplicate runs were performed at 17°C using a plasma‐derived model matrix and PRV; the final S/D mix concentrations were 5% of manufacturing. Asterisks indicate viral inactivation below the detection limit. C, D, Duplicate runs were performed at 1°C using a recombinant model matrix and Xenotropic murine leukemia virus (X‐MuLV); the final S/D mix concentrations were 10% of manufacturing. *SD* only shown if larger than the height of symbols). PRV, pseudorabies virus; RF, reduction factor; S/D, solvent/detergent; *SD*, standard deviation; TNBP, tri‐n‐butyl‐phosphate; TX‐100, Triton X‐100

Under all conditions tested **VI** showed equivalent virus inactivation to TX‐100 (**I**) (Figure 5). In stark contrast, compound **VII**, despite its similarity to **VI**, inactivated less efficiently at warm temperatures and was even less potent at cold conditions.

The crucial finding that the phenol polyethylene glycol connection in TX‐100 (**I**) can be replaced by another linkage such as a benzyl ether while fully preserving virus inactivation activity, corresponded to our aim of removing the phenol group. This undesired group is in fact the root cause for the estrogen receptor‐binding (i.e., pseudo‐hormone) activity of TX‐100 (**I**) degradation products ^4^, ^16^ in certain aquatic organisms.

This finding reveals that not only phenol but also other benzyl alcohol derivatives might be used to connect the hydrophilic part to the lyophilic part of the detergent. Surprisingly, a seemingly insignificant shortening of the alkyl chain had a pronounced negative effect on the virus inactivation activity of the corresponding compound.

### 3.7 Sar#5: Modification of the alky side chain

**Scheme 5:**
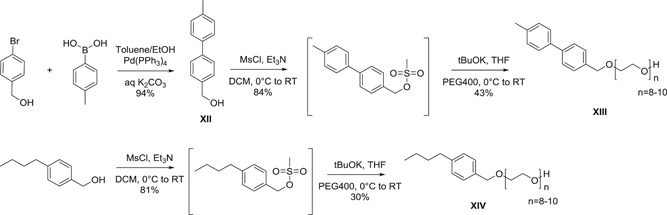
Synthesis route of detergents **XIII** and **XIV**

To determine whether the length of the lipophilic part was also key to activity, compounds **XIII** and **XIV** were synthesized. Although *n*‐butyl benzyl alcohol could be purchased from a chemical supplier, benzyl alcohol **XII**, with a biphenyl structure had to be prepared by an uneventful and high yielding (94%) Suzuki coupling step. Following standard protocol, both detergents **XIII** and **XIV** were obtained in gram quantities in respectively 34% and 24% overall yield (Scheme 5).

**Figure 6:**
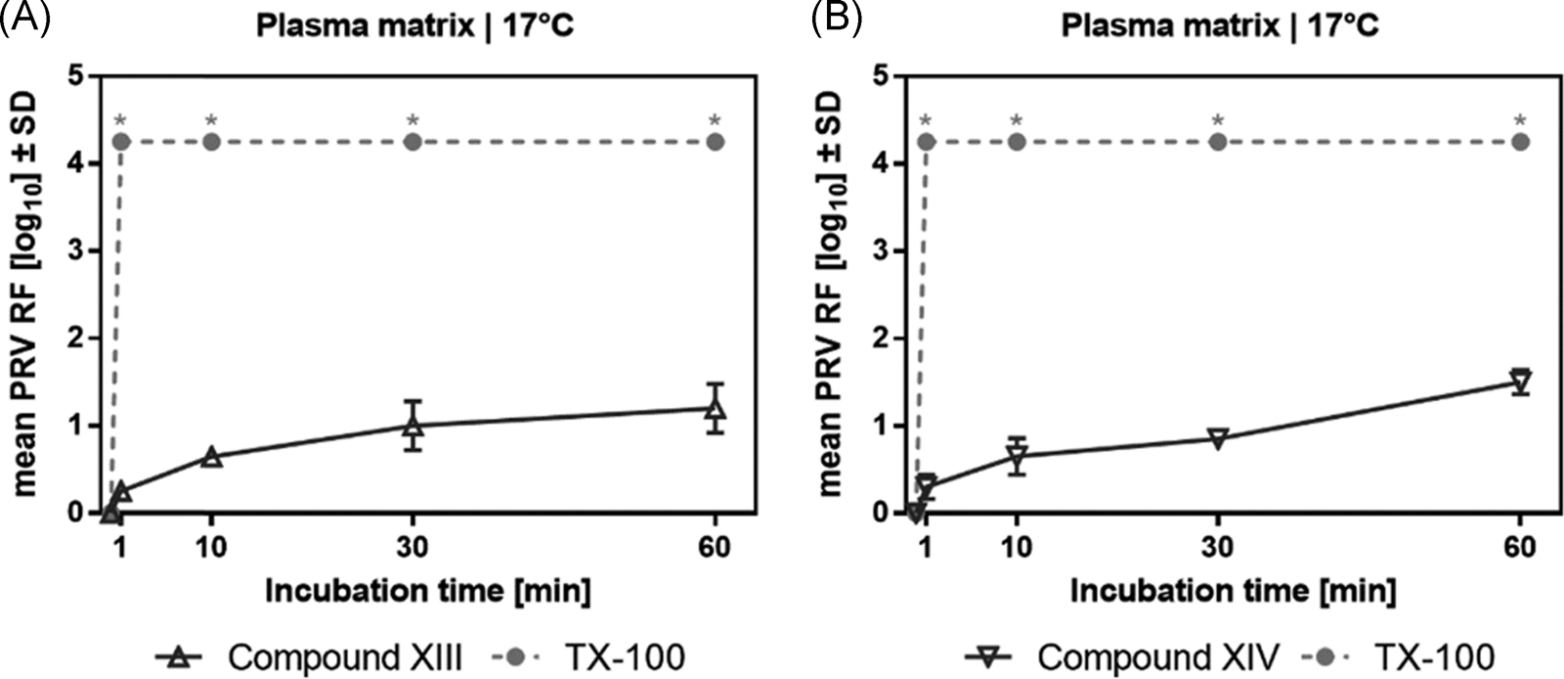
Virus inactivation by S/D mixes containing novel compounds XIII and XIV. Duplicate runs were performed at 17°C using a plasma‐derived model matrix and PRV. PS80 and TNBP were combined with (A) compound XIII or (B) compound XIV and added to the virus‐spiked matrix to reach a final concentration of 5% as specified for manufacturing. Samples were drawn 1, 10, 30, and 60 min after S/D mix addition; viral loads of these samples were compared to a sample drawn before S/D mix addition to calculate the respective RF. Asterisks indicate viral inactivation below detection limit. *SD* only shown if larger than the height of symbols). PRV, pseudorabies virus; RF, reduction factor; S/D, solvent/detergent; *SD*, standard deviation; TNBP, tri‐n‐butyl‐phosphate; TX‐100, Triton X‐100

According to measurement calculations, the two detergents **XIII** and **XIV** have a very similar length for *n* = 9 (42.9 and 40.6 Å, respectively) compared to **VI** and TX‐100 (**I**) (39.5 and 39.6 Å, respectively). However, virus inactivation studies at standard temperature revealed unsatisfactory potency of both compounds (Figure 6), and therefore studies under cold conditions were not performed. Apparently, adding aromatic density to the detergent or removing the tetramethyl substitution of the alkyl chain drastically impaired the ability to destroy the viral envelope, even though the length ratio of hydrophilic part to hydrophobic part was maintained.

Detergent **VI** has several advantages over the other synthesized detergents from this library, as an alternative to TX‐100 (**I**). Firstly, **VI** showed effective and reliable virus inactivation for the process matrices and viruses tested. Secondly, its very similar structure to TX‐100 (**I**) suggests that also the chemo‐physical properties of the detergent (foaming, critical micelle concentration, solubility, detectability), as well as interaction with the protein(s) of interest will be highly similar. This should ensure that changes in the affected processes, such as analytical method or removal process, will be only minimal. Finally, from an ecological point of view, after release of compound **VI** into the environment, the polyethylene glycol chain is broken down in a similar manner as for TX‐100; however, the revealed benzyl alcohol will be easily oxidized to the corresponding benzoic acid,^17^, ^18^, ^19^ which is expected to interact less with estrogen receptors in animal toxicology studies than the corresponding octyl‐phenol, as this metabolite has a completely different polarity and geometric structure than the phenol derivatives produced during TX‐100 (**I**) degradation. In addition, further differences between phenols et benzyl alcohols in their abilities to be metabolized either through oxidative or bacterial processes might favor benzylic compounds in regard to the impact on their biodegradations.^20^, ^21^

### 3.8 Scalable synthesis of VI

**Scheme 6:**
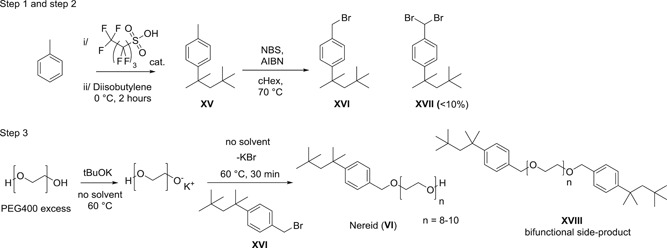
Optimized 3‐step synthesis of detergent **VI**

The laboratory‐scale 6‐step synthesis (Scheme 3) yields hundreds of grams of detergent within a couple of months, however, this route utilizes 4 chromatography steps as well as expensive (Tf_2_O, Pd catalyst), toxic (octylphenol, Zn(CN)_2_, DMF, MsCl), and hazardous (LiAlH_4_) chemicals. Moreover, as the demand for material grew rapidly to accommodate feasibility studies, the synthetic route was upgraded to a convenient 3‐step synthesis using cheap, nontoxic and safe starting materials available in bulk (Scheme 6). The work‐up was optimized to ultimately replace the chromatography steps by convenient large‐scale work‐up procedures which reduced the volume of required solvents.

#### 3.8.1 Step 1

Taking advantage of the inducing effect of the methyl group of toluene to achieve the para‐substitution of the target molecule, an acid‐catalyzed Friedel‐Craft alkylation using diisobutylene was envisaged to access the hydrocarbon **XV**. The original sulfuric acid was replaced by a more soluble nonafluoro‐1‐butanesulfonic acid. Due to the bulkiness of the electrophile generated from the diisobutylene as a mixture 2,4,4‐trimethyl‐1‐pentene/2,4,4‐trimethyl‐2‐pentene (3:1), no ortho product was detected. The work‐up included a simple quench of the acid followed by extractive aqueous washes. Other isomers originating from likely cation rearrangements of the alkyl chain were also formed and could be removed entirely during the purification of the following Step 2. Exhaustive evaporation of the solvent is needed to remove the totality of the toluene, as it might react competitively with the NBS reagent during the next step.

#### 3.8.2 Step 2

The crude oily hydrocarbon **XV** was then engaged at 70°C with AIBN and NBS to form benzyl bromide **XVI** in a radical bromination in cyclohexane, during which after radical propagation, succinimide precipitates as a white solid. After filtration and concentration, the crude product containing some starting material as well as dibrominated product **XVII** was precipitated in IPA to yield a product with an approximate 90% purity. Dibrominated product **XVII** was synthesized separately to confirm the identity of this main side‐product. Further two recrystallizations in IPA delivered the benzyl bromide intermediate **XVI** in moderate yield over two steps (25%–30%) but at >99% purity (200 nm). Thus, all side products from the first and second step could be removed and a crystalline colorless solid (large plates) was obtained after drying.

#### 3.8.3 Step 3

Without solvent, a 5‐molar excess of PEG400 (polyethylene glycol with an average MW of 380–420 g/mol) was deprotonated at 60°C by addition of tBuOK. To the mono‐deprotonated PEG400, the benzyl bromide intermediate **XVI** was added in one portion before the reaction mixture was cooled 30 min later by addition of ice/water followed by pH adjustment, removal of the discoloration, and addition of EtOAc. Several aqueous washes assured that the excess of PEG400 was entirely removed. After concentration of the EtOAc, the residue containing some bi‐functional product **XVIII** was re‐dissolved in EtOH/water and non‐polar washes were performed with cyclohexane to remove a large portion of the bi‐functional product **XVIII**. An independent synthesis of bifunctional side‐product **XVIII** confirmed the structure and served as reference material. A final concentration delivered several hundred grams of product **VI** as a yellowish clear oil with a purity of >99% (LC‐ELSD). It can be noted that the route utilizes only five solvents (toluene, cHex, IPA, EtOAc, and EtOH), all of them being nonhazardous and recoverable after concentration.

This material is currently in use for feasibility studies, engineering and confirmation runs. Further chemical development studies and improvements are in progress to continuously optimize the process and prepare for large‐scale industrial production of this detergent, which was named “Nereid” after Amphitrite, the Greek sea goddess and wife of Poseidon but also the mother of Triton, from whom comes the prefix “amphi‐“ (ἀμφί) meaning “both kinds, on both sides” used in modern words such as amphibious, amphitheater or amphiphile (detergent).

## 4 CONCLUSIONS

The limited number of commercially available detergents that meet the pre‐defined structural requirements of similarity with TX‐100 (**I**) dramatically narrowed the screening possibilities described in this study. In this regard, the plan arose to develop a tailor‐made detergent that fulfills the prerequisites of the virus inactivation potency of TX‐100 (**I**) without being harmful to the environment. Structure‐function analyses indicated that the alkyl chain substitution must follow the 1,1,3,3‐tetramethylbutyl pattern of TX‐100 (**I**) and must be connected to an aromatic phenyl ring to retain full virus inactivation properties. The installation of a –CH_2_– between the aromatic ring and the polyethylene glycol chain resulted in conservation of the inactivation potential, thus the phenol‐free detergent Nereid was discovered. A convenient 3‐step synthesis was developed, which allows the production of hundreds of grams of pure detergent using reliable protocols and standard equipment. Further work is in progress to adapt the synthesis route to the multi‐kg, and ultimately ton scales. Investigations are underway to analyze and dismantle the exact molecular mechanism of the S/D treatment. Although protein compatibility as well as process compatibility have been verified on a considerable range of products (gene therapy product, recombinant, and plasma‐based biologics),^11^ extended studies such as toxicity studies have recently been initiated.

## CONFLICT OF INTERESTS

Jean‐Baptiste Farcet, Richard Scheinecker, and Otto Kostner are employees of Baxalta Innovations GmbH, Vienna, Austria, now part of the Takeda group of companies. Johanna Kindermann, Michael Karbiener, and Thomas R. Kreil are employees of Baxter AG, Vienna, Austria, now part of the Takeda group of companies. Jean‐Baptiste Farcet, Michael Karbiener, and Thomas R. Kreil are Takeda stock owners. Jean‐Baptiste Farcet, Johanna Kindermann, and Thomas R. Kreil are inventors of a submitted patent application related to Brij C10, Triton X‐100 reduced, and Nereid.

## AUTHOR CONTRIBUTIONS

Jean‐Baptiste Farcet conceived the Sar study, designed and synthesized the new compounds. Jean‐Baptiste Farcet, Richard Scheinecker, and Otto Kostner analyzed the new chemicals. Jean‐Baptiste Farcet and Otto Kostner optimized the synthesis of Nereid. Johanna Kindermann planned and coordinated the virus inactivation experiments, Michael Karbiener gave conceptional input and structured the manuscript. Thomas R. Kreil initiated and coordinated the study. Jean‐Baptiste Farcet, Michael Karbiener, and Thomas R. Kreil contributed to the writing of the manuscript.

## Supporting information

Supplementary information.

## Data Availability

The data that supports the findings of this study are available in the supplementary material of this article.
