# Supplementary material for: Synthesis of “Nereid,” a new phenol‐free detergent to replace Triton X‐100 in virus inactivation
Source: J Med Virol. 2020 Dec 17;93(6):3880–9. doi: 10.1002/jmv.26708 (PMC8247325; doi:10.1002/jmv.26708)

**Supplementary Information**

**Synthesis of ‘Nereid’, a new phenol-free detergent to replace Triton X-100 in virus inactivation processes**

Jean-Baptiste Farcet^1^, Johanna Kindermann^2^, Michael Karbiener^2^, Richard Scheinecker^1^, Otto Kostner^1^, Thomas R. Kreil^2^

^1^Pharmaceutical Sciences, Baxalta Innovations GmbH, Vienna, now part of the Takeda group of companies

^2^Global Pathogen Safety, Baxter AG, Vienna, now part of the Takeda group of companies

**Correspondance:**

Jean-Baptiste Farcet, Takeda, Industriestrasse 131, 1221 Vienna, Austria

phone: +43-1-20100-247-6917

E-mail: jean-baptiste.farcet@takeda.com

Table of Contents

[Graphical Abstract 4](#_Toc21613601)

[Keywords 4](#_Toc21613602)

[Material 5](#_Toc21613603)

[Synthesis methods: 5](#_Toc21613604)

[Synthetic procedures 6](#_Toc21613605)

[Synthesis of 3,5,5-trimethyl-1-hexanol polyethoxylate (**III**) 6](#_Toc21613606)

[Synthesis of Geraniol polyethoxylate (**IV**) 8](#_Toc21613607)

[Synthesis of 3,7-dimethyloctanol polyethoxylate (**V**) 9](#_Toc21613608)

[Synthesis of intermediate **VIII** 10](#_Toc21613609)

[Synthesis of intermediate **IX** 11](#_Toc21613610)

[Synthesis of intermediate **X** 12](#_Toc21613611)

[Synthesis of intermediate **XI** 13](#_Toc21613612)

[Synthesis of **VI** from **XI** 14](#_Toc21613613)

[Synthesis of *tert*-Butylbenzylalcohol polyethoxylate (**VII**) 16](#_Toc21613614)

[(4'-methyl-[1,1'-biphenyl]-4-yl) methanol (**XII**) 17](#_Toc21613615)

[Synthesis of (4'-methyl-[1,1'-biphenyl]-4-yl)methanol polyethoxylate (**XIII**) 18](#_Toc21613616)

[Synthesis of polyethoxylated (4-butylphenyl)methanol (**XIV**) 20](#_Toc21613617)

[Synthesis of 1-methyl-4-(2,4,4-trimethylpentan-2-yl)benzene (**XV**) 22](#_Toc21613618)

[Synthesis of 1-(bromomethyl)-4-(2,4,4-trimethylpentan-2-yl)benzene (**XVI**) 23](#_Toc21613619)

[Synthesis of the side product **XVII** 24](#_Toc21613620)

[Synthesis of **VI** from **XVI** 25](#_Toc21613621)

[Synthesis of the side product bi-functional product **XVIII** 26](#_Toc21613622)

[NMR Spectra 27](#_Toc21613623)

[^1^H-NMR spectra of compound **III** 27](#_Toc21613624)

[^13^C-NMR-Jmod spectra of compound **III** 27](#_Toc21613625)

[^1^H-NMR spectra of compound **IV** 28](#_Toc21613626)

[^13^C-NMR-Jmod spectra of compound **IV** 28](#_Toc21613627)

[^1^H-NMR spectra of compound **V** 29](#_Toc21613628)

[^13^C-NMR-Jmod spectra of compound **V** 29](#_Toc21613629)

[^1^H-NMR spectra of compound **VIII** 30](#_Toc21613630)

[^13^C-NMR-Jmod spectra of compound **VIII** 30](#_Toc21613631)

[^1^H-NMR spectra of compound **IX** 31](#_Toc21613632)

[^13^C-NMR-Jmod spectra of compound **IX** 31](#_Toc21613633)

[^1^H-NMR spectra of compound **X** 32](#_Toc21613634)

[^13^C-NMR-Jmod spectra of compound **X** 32](#_Toc21613635)

[^1^H-NMR spectra of compound **XI** 33](#_Toc21613636)

[^13^C-NMR-Jmod spectra of compound **XI** 33](#_Toc21613637)

[^1^H-NMR spectra of compound **VI** 34](#_Toc21613638)

[^13^C-NMR-Jmod spectra of compound **VI** 34](#_Toc21613639)

[^1^H-NMR spectra of compound **VII** 35](#_Toc21613640)

[^13^C-NMR-Jmod spectra of compound **VII** 35](#_Toc21613641)

[^1^H-NMR spectra of compound **XII** 36](#_Toc21613642)

[^13^C-NMR-Jmod spectra of compound **XII** 36](#_Toc21613643)

[^1^H-NMR spectra of compound **XIII** 37](#_Toc21613644)

[^13^C-NMR-Jmod spectra of compound **XIII** 37](#_Toc21613645)

[^1^H-NMR spectra of compound **XIV** 38](#_Toc21613646)

[^13^C-NMR-Jmod spectra of compound **XIV** 38](#_Toc21613647)

[^1^H-NMR spectra of compound **XV** 39](#_Toc21613648)

[^13^C-NMR-Jmod spectra of compound **XV** 39](#_Toc21613649)

[^1^H-NMR spectra of compound **XVI** 40](#_Toc21613650)

[^13^C-NMR-Jmod spectra of compound **XVI** 40](#_Toc21613651)

[^1^H-NMR spectra of compound **XVII** 41](#_Toc21613652)

[^13^C-NMR-Jmod spectra of compound **XVII** 41](#_Toc21613653)

[^1^H-NMR spectra of compound **XVIII** 42](#_Toc21613654)

[^13^C-NMR-Jmod spectra of compound **XVIII** 42](#_Toc21613655)

# Graphical Abstract


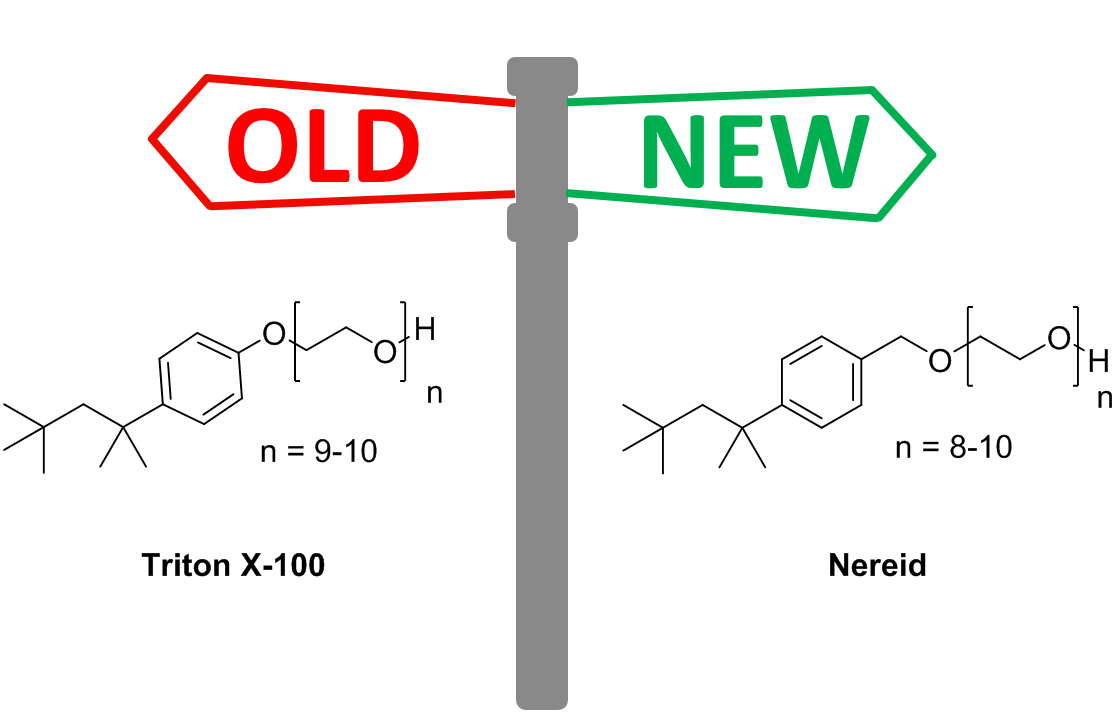


# Keywords

detergent; virus inactivation; Triton X-100; organic synthesis; structure-activity relationship

# Material

All the syntheses were carried out with commercially available reagents and solvents (Merck, Karl Roth, TCI, WVR) used without further purification.

# Synthesis and analytic methods:

^1^H and ^13^C-NMR spectra were recorded on a Bruker AV600 spectrometer at room temperature using standard pulse programs. Chemical shifts (*δ*) are quoted in parts per million (ppm) and referenced to the appropriate residual solvent signal. Coupling constants (*J*) are reported to the nearest 0.1 Hz. LC-Mass spectra (m/z) were run on a Shimadzu LC-MS 2020, HRMS (High resolution mass) analysis was performed on MicroMass TOF spectrometer from Waters (ESI+). Flash chromatography was performed on silica gel (Merck Kieselgel 60, 0.040 - 0.063 mm). Thin Layer Chromatography (TLC) was performed using TLC Silica Gel 60 F24 (aluminium sheets from Merck). Analytical HPLC chromatograms were run in an Agilent System equipped with DAD and/or ELSD detector. Melting points were measured on a DSC instrument (DSC 2000 from TA Instruments) or on a microscope Reichert Thermovar. FT-IR (ATR) was measured on a Bruker tensor 27 spectrometer equipped with an ATR BioATR cell II using neat samples (for liquid and oily compounds) or using dichloromethane dissolved solution samples (for solid samples) measured after full evaporation of the volatiles. Calculations of molecule lengths were performed using the Software Chem3D V15.1.

# Synthetic procedures

## Synthesis of 3,5,5-trimethyl-1-hexanol polyethoxylate (**III**)

3,5,5-trimethyl-1-hexanol (3.0 g, 20.8 mmol) was dissolved in anhydrous CH_2_Cl_2_ (70 mL) and cooled in an ice/water bath. MsCl (1.93 mL, 25.0 mmol) was added slowly followed by the dropwise addition of NEt_3_ (3.50 mL, 25 mmol). The reaction warmed slowly to ambient temperature and was further stirred overnight. An aqueous saturated NaHCO_3_ solution (75 mL) was added as well as EtOAc (180 mL) and the reaction mixture was agitated vigorously. The aqueous phase was extracted with EtOAc (60 mL) and the combined organic phases were washed repeatedly with water (50 mL) and brine (75 mL). After drying and filtration, the organic phase was then concentrated in vacuo to yield 4.5 g (Yield: 98%) of a clear oil, that was engaged directly in the next step. R_f_ = 0.54 (toluene/EtOAc 9:1).

PEG400 (10.8 g, 27.0 mmol) was dissolved in anhydrous THF (180 mL) under Argon and the mixture was cooled in an ice/water bath. tBuOK (3.0 g, 27.0 mmol) was added portion wise over 3 min and the mixture was stirred 60 min in the cool bath. In the meantime, the Mesylate intermediate (2.0 g, 9.0 mmol) was suspended in THF (10 mL) and the solution was added to the cooled deprotonated PEG400 solution over 4 min. The reaction was warmed slowly to ambient temperature and further stirred overnight. The reaction mixture was filtered over a pad of Celite and the cake was rinsed with CH_2_Cl_2_ (10 mL). The reaction mixture was concentrated, and the residue was dissolved in EtOAc (150 mL). The solution was transferred into a separatory funnel and the organic phase was washed successively with water/brine (1:1; 2 x 50 mL) and finally dried over MgSO_4_ to yield 3.8 g of crude oily residue. This residue was charged on top of a SiO_2_ column and eluted with CH_2_Cl_2_/MeOH (0 to 5%). Concentration of the pure fractions yielded 3.17 g (Yield: 65%) of a pale-yellow oil. R_f_ = 0.51 (CH_2_Cl_2_/MeOH 10:1). ^1^H-NMR (600 MHz, CDCl_3_): *δ* = 3.78-3.50 (m, 32.7H), 3.47 (t, *J* = 6.7 Hz, 2H), 2.13 (brs, OH), 1.61-1.53 (m, 2H), 1.47-1.38 (m, 1H), 1.21 (dd, *J_1_* = 3.2 Hz, *J_2_* = 14.0 Hz, 1H), 1.04 (dd, *J_1_* = 6.1 Hz, *J_2_* = 14.0 Hz, 1H), 0.91 (d, *J* = 6.6 Hz, 3H), 0.88 (s, 9H). ^13^C-NMR (150 MHz, CDCl_3_): *δ* = 72.7, 70.8, 70.7 (m), 70.5, 70.2, 70.0, 61.9, 51.3, 39.1, 30.1, 26.4, 23.0. IR (cm^-1^): 2953, 2866, 1468, 1351, 1249, 1104, 1038, 949, 670, 588.HRMS (ESI^+^) (m/z): [M+Na]^+^ calculated for C_27_H_57_NaO_10_ (n=9): 563.3771; found: 563.3793.

## Synthesis of Geraniol polyethoxylate (**IV**)

Geraniol (3.0 g, 19.1 mmol) was dissolved in anhydrous CH_2_Cl_2_ (100 mL) and cooled in an ice/water bath. NEt_3_ (3.4 mL, 22.9 mmol) was added slowly followed by the dropwise addition of MsCl (1.78 mL, 22.9 mmol). The reaction was further stirred 25 min at ambient temperature. An aqueous saturated NaHCO_3_ solution (100 mL) was added as well as EtOAc (250 mL) and the reaction mixture was agitated vigorously. The aqueous phase was extracted with EtOAc (50 mL) and the combined organic phases were washed repeatedly with water (100 mL) and brine (100 mL). After drying and filtration, the organic phase was then concentrated in vacuo to yield 3.2 g (Yield: 72%) of a clear oil (prone to degradation), that was engaged directly in the next step.

PEG400 (10.1 g, 24 mmol) was dissolved in anhydrous THF (60 mL) under Argon and the mixture was cooled in an ice/water bath. tBuOK (2.67 g, 23.8 mmol) was added portion wise over 3 min and the mixture was stirred 60 min in the cool bath. In the meantime, the Mesylate intermediate (1.85 g, 8.0 mmol) was suspended in THF (15 mL) and the solution was added to the cooled deprotonated PEG400 solution over 4 min. The reaction was warmed slowly to ambient temperature and further stirred overnight. The reaction mixture was filtered over a pad of Celite and the cake was rinsed with CH_2_Cl_2_ (10 mL). The reaction mixture was concentrated, and the residue was dissolved in EtOAc (250 mL) and water (150 mL). The solution was transferred into a separatory funnel and the organic phase was washed successively with water (2 x 80 mL) and brine (50 mL) and finally dried over MgSO_4_ to yield 3.2 g of crude oily residue. This residue was charged on top of a SiO_2_ column and eluted with CH_2_Cl_2_/MeOH (0 to 5%). Concentration of the pure fractions yielded 2.26 g (Yield: 53%) of a pale-yellow oil. R_f_ = 0.29 (CH_2_Cl_2_/MeOH 20:1). ^1^H-NMR (600 MHz, CDCl_3_): *δ* = 5.37-5.31 (m, 1H), 5.11-5.05 (m, 1H), 4.03 (d, *J* = 6.8 Hz, 2H), 3.80-3.50 (m, 41.2H), 2.30 (brs, OH), 2.12-1.99 (m, 4H), 1.67 (s, 3H), 1.65 (s, 3H), 1.59 (s, 3H). ^13^C-NMR (150 MHz, CDCl_3_): *δ* = 140.2, 131.8, 124.1, 121.0, 72.7, 70.8, 70.7 (m), 70.5, 69.2, 67.8, 61.9, 39.7, 26.5, 25.9, 17.8, 16.6. IR (cm^-1^): 2865, 1453, 1350, 1100, 948, 736, 667, 650, 631, 544. HRMS (ESI^+^) (m/z): [M+Na]^+^ calculated for C_28_H_54_NaO_10_ (n=9): 573.3615; found: 573.3624.

## Synthesis of 3,7-dimethyloctanol polyethoxylate (**V**)

3,7-dimethyloctanol (3.0 g, 18.6 mmol) was dissolved in anhydrous CH_2_Cl_2_ (75 mL) and cooled in an ice/water bath. NEt_3_ (3.15 mL, 22.3 mmol) was added slowly followed by the dropwise addition of MsCl (1.75 mL, 22.3 mmol). The reaction was further stirred 120 min at ambient temperature. An aqueous saturated NaHCO_3_ solution (85 mL) was added as well as EtOAc (150 mL) and the reaction mixture was agitated vigorously. The aqueous phase was extracted with EtOAc (150 mL) and the combined organic phases were washed repeatedly with water (100 mL) and brine (50 mL). After drying and filtration, the organic phase was then concentrated in vacuo to yield 4.3 g (Yield: 98%) of a clear oil that was engaged directly in the next step.

PEG400 (18.0 g, 44.4 mmol) was dissolved in anhydrous THF (120 mL) under Argon and the mixture was cooled in an ice/water bath. tBuOK (4.97 g, 44.4 mmol) was added portion wise over 3 min and the mixture was stirred 60 min in the cool bath. In the meantime, the Mesylate intermediate (3.52 g, 14.8 mmol) was suspended in THF (15 mL) and the solution was added to the cooled deprotonated PEG400 solution over 4 min. The reaction was warmed slowly to ambient temperature and further stirred overnight. The reaction mixture was filtered over a pad of Celite and the cake was rinsed with CH_2_Cl_2_ (10 mL). The reaction mixture was concentrated, and the residue was dissolved in EtOAc (150 mL) and water (100 mL). The solution was transferred into a separatory funnel and the organic phase was washed successively with water (2 x 80 mL) and brine (50 mL) and finally dried over MgSO_4_ to yield 8.5 g of crude oily residue. This residue was charged on top of a SiO_2_ column and eluted with CH_2_Cl_2_/MeOH (0 to 5%). Concentration of the pure fractions yielded 5.91 g (Yield: 74%) of a pale-yellow oil. R_f_ = 0.42 (CH_2_Cl_2_/MeOH 20:1). ^1^H-NMR (600 MHz, CDCl_3_): *δ* = 3.80-3.51 (m, 41.7H), 3.51-3.42 (m, 2H), 2.20 (brs, OH), 1.66-1.58 (m, 1H), 1.55-1.46 (m, 2H), 1.41-1.33 (m, 1H), 1.33-1.18 (m, 3H), 1.16-1.06 (m, 3H), 0.86 (d, *J* = 6.5 Hz, 3H), 0.85 (d, *J* = 6.5 Hz, 3H), 0.86 (d, *J* = 6.5 Hz, 3H). ^13^C-NMR (150 MHz, CDCl_3_): *δ* = 72.7, 70.8, 70.7 (m), 70.4, 70.2, 70.0, 61.9, 39.4, 37.5, 36.7, 30.0, 28.1, 24.8, 22.9, 22.8, 19.8. IR (cm^-1^): 2954, 2922, 2867, 1738, 1461, 1352, 1247, 1102, 1037, 943, 669, 650. HRMS (ESI^+^) (m/z): [M+Na]^+^ calculated for C_28_H_58_NaO_10_ (n=9): 577.3928; found: 577.3931.

## Synthesis of intermediate **VIII**

Octyl-phenol (170.3 g, 800 mmol) was placed in a 3 neck 2 L flask equipped with an inner thermometer and stirring bar. Anhydrous CH_2_Cl_2_ (1000 mL) was added to the flask and stirring was started. After dissolution of the starting material the solution was cooled to 0°C. After total dissolution NEt_3_ (225 mL, 1.6 mol) was added within 10 min. A solution Tf_2_O (256 g, 907 mmol) in CH_2_Cl_2_ (180 mL) was added at 0°C to the reaction mixture over 120 min and the reaction was stirred overnight at room temperature. An aqueous saturated NaHCO_3_ solution (400 mL) was added and the reaction mixture was extracted. The organic phase was washed repeatedly with water (2 x 400 mL) and brine (500 mL). The organic phase was then concentrated in vacuo. Toluene (300 mL) was added to the residue and the crude product was concentrated to yield 314 g of crude black liquid. This residue was charged on top of a SiO_2_ plug and eluted with petroleum ether/EtOAc (0 to 2%). Concentration of the pure fractions yielded 269.5 g (Yield: 99.6%) of a clear colorless oil. R_f_ = 0.74 (petroleum ether/EtOAc 5%). ^1^H-NMR (600 MHz, CDCl_3_): *δ* = 7.43 (d, *J* = 9.0 Hz, 2H), 7.17 (d, *J* = 9.0 Hz, 2H), 1.74 (s, 2H), 1.37 (s, 6H), 0.71 (s, 9H). ^13^C-NMR (150 MHz, CDCl_3_): *δ* = 151.0, 147.5, 128.1 (2C), 120.6 (2C), 118.9 (d, *J* = 321 Hz, 1C), 57.2, 38.8, 32.5, 32.0 (3C), 31.7 (2C). IR (cm^-1^): 2954, 2909, 2875, 1502, 1423, 1249, 1209, 1141, 1015, 886, 839, 667, 633.

## Synthesis of intermediate **IX**

Triflate **VIII** (269 g, 795 mmol) was dissolved in anhydrous and degassed DMF (1.3 L) and Zn(CN)_2_ (95.3 g, 795 mmol) and Pd(PPh_3_)_4_ (25 g, 21.5 mmol) were added sequentially. The reaction mixture was warmed to 80°C for 3 hrs followed by removal of the DMF under vacuum. Toluene (300 mL) was added to the residue and the crude product was concentrated to yield 432 g of black residue. This crude product was charged on top of a SiO_2_ plug and eluted with petroleum ether/EtOAc (0 to 10%). Concentration of the pure fractions yielded 128.1 g (Yield: 74.8%) of a clear colorless oil that crystallized upon standing at 2-8°C. R_f_ = 0.23 (petroleum ether/EtOAc 2%). m.p. = 24-26°C. ^1^H-NMR (600 MHz, CDCl_3_): *δ* = 7.57 (d, *J* = 8.6 Hz, 2H), 7.47 (d, *J* = 8.6 Hz, 2H), 1.77 (s, 2H), 1.37 (s, 6H), 0.71 (s, 9H). ^13^C-NMR (150 MHz, CDCl_3_): *δ* = 156.2, 131.8 (2C), 127.1 (2C), 119.3, 109.2, 56.8, 39.3, 32.5, 31.9 (3C), 31.3 (2C). IR (cm^-1^): 2958, 2898, 2869, 2227, 1607, 1506, 1471, 1401, 1366, 1248, 1094, 835, 667. HRMS (ESI^+^) (m/z): [M+H]^+^ calculated for C_15_H_22_N: 216.1752; found: 216.1750.

## Synthesis of intermediate **X**

Nitrile **IX** (127.6 g. 592.5 mmol) dissolved in MeOH (500 mL), aqueous NaOH 4 M (750 mL) was added and the mixture was brought to reflux and kept at this temperature (80°C) overnight. An additional aqueous NaOH 10 M (150 mL) was added to the warm mixture and the solution was heated further for 20 hrs. After cooling to ambient temperature, the content of the reaction vessel was transferred into a large Beaker and cooled in an ice bath. Aqueous HCl 4 M (1.1 L) was added within 30 min, at this point the pH was acidic as indicated by pH paper and a white solid had precipitated. The precipitate was filtered and rinsed with water (500 mL). The wet cake was transferred to a 2 L flask and dried under vacuum for 3 days to yield 127.5 g (Yield: 91.9%) of a white powder. R_f_ = 0.42 (petroleum ether/EtOAc 2:1). m.p. (DSC) = 141.3°C. ^1^H-NMR (600 MHz, CDCl_3_): *δ* = 8.00 (d, *J* = 9.8 Hz, 2H), 7.48 (d, *J* = 9.8 Hz, 2H), 1.79 (s, 2H), 1.40 (s, 6H), 0.72 (s, 9H). ^13^C-NMR (150 MHz, CDCl_3_): *δ* = 172.2, 157.1, 130.0 (2C), 126.5 (2C), 126.5, 57.0, 39.3, 32.6, 31.9 (3C), 31.5 (2C). IR (cm^-1^): 2954, 1676, 1610, 1420, 1288, 958, 853, 780, 709, 667, 642.HRMS (ESI^+^) (m/z): [M+H]^+^ calculated for C_15_H_23_O_2_: 235.1698; found: 235.1702.

## Synthesis of intermediate **XI**

Carboxylic acid **X** (127 g, 542 mmol) was suspended in dried mTHF (1.2 L) and cooled to -10°C. A solution of LiAlH_4_ in THF (1 M, 575 mL, 575 mmol) was added over 60 min, then the reaction was warmed slowly to ambient temperature and further stirred overnight. The content of the reaction vessel was transferred into a large Beaker and the excess of hydride was carefully quenched with ice (15 g). Aqueous HCl 3 M (500 mL) was added within 20 min, at this point the pH was acidic as indicated by pH paper. EtOAc (300 mL) was added to the crude mixture and the 2 phases were vigorously agitated. The aqueous phase was back extracted twice with EtOAc (300 mL and 500 mL). The combined organic phases were washed successively with an aqueous saturated NaHCO_3_ solution (300 mL), water (300 mL) and brine (500 mL). The organic phase was then concentrated in vacuo. Toluene (300 mL) was added to the residue and the crude product was concentrated to yield 123.3 g of a clear yellowish oil. This residue was charged on top of a SiO_2_ plug and eluted with petroleum ether/EtOAc (0 to 15%). Concentration of the pure fractions yielded 85.3 g (Yield: 71.4%) of an amorphous white solid. R_f_ = 0.37 (petroleum ether/EtOAc 4:1). m.p. (DSC) = 176.8°C. ^1^H-NMR (600 MHz, CDCl_3_): *δ* = 7.37 (d, *J* = 8.4 Hz, 2H), 7.27 (d, *J* = 8.4 Hz, 2H), 4.66 (s, 2H), 1.75 (s, 2H), 1.68 (br s, 1H), 1.36 (s, 6H), 0.71 (s, 9H). ^13^C-NMR (150 MHz, CDCl_3_): *δ* = 149.9, 137.8, 126.7 (2C), 126.4 (2C), 65.4, 57.0, 38.6, 32.5, 31.9 (3C), 31.7 (2C). IR (cm^-1^): 2954, 2901, 2868, 1515, 1467, 1365, 1251, 1213, 1094, 1014, 817. HRMS (ESI^+^) (m/z): [M-H_2_O+H]^+^ calculated for C_15_H_23_: 203.1799; found: 203.1804.

## Synthesis of **VI** from **XI**

Benzyl alcohol **XI** (84.8 g, 385 mmol) was dissolved in anhydrous CH_2_Cl_2_ (1 L) and cooled in an ice/water bath. NEt_3_ (110 mL, 770 mmol) was added followed by the slow addition of a solution of MsCl (45 mL, 577 mmol) in anhydrous CH_2_Cl_2_ (25 mL) over 60 min. The reaction warmed slowly to ambient temperature and was further stirred overnight. An aqueous saturated NaHCO_3_ solution (420 mL) was added and the reaction mixture was agitated vigorously. The organic phase was washed repeatedly with water (2 x 500 mL) and brine (300 mL). The organic phase was then concentrated in vacuo. Toluene (200 mL) and CH_2_Cl_2_ (100 mL) were added to the residue and the crude product was concentrated to yield 90 g (Yield: 78.4%) of an orange semi solid, which was engaged directly in the next step. R_f_ = 0.71 (petroleum ether/EtOAc 4:1).

PEG400 (360 g, 900 mmol) was dissolved in anhydrous THF (1 L) and tBuOK (90 g, 802 mmol) was added at ambient temperature portion wise over 15 min and the mixture was stirred 60 min at ambient temperature and cooled in an ice bath. In the meantime, Mesylate (89.5 g, 300 mmol) was suspended in THF (300 mL) and the milky orange solution was added to the cooled deprotonated PEG400 solution over 20 min. The reaction was warmed slowly to ambient temperature and further stirred overnight. Ice (500 g) was added as well as aqueous HCl 1 M (820 mL). The THF was removed under vacuum and EtOAc (1 L) was added. The phases were agitated, and the organic phase was washed successively with water (2 x 500 mL). Each aqueous phase was back extracted with EtOAc (300 mL). The combined organic phases were washed with water (500 mL) and concentrated in vacuo. Toluene (250 mL) was added to the residue and the crude product was concentrated to yield 125.2 g of a clear yellowish oil. This residue was charged on top of a SiO_2_ plug and eluted with CH_2_Cl_2_/MeOH (0 to 8%). Concentration of the pure fractions yielded 107.5 g (Yield: 59.5%) of a light brown clear oil. R_f_ = 0.37-0.22 (CH_2_Cl_2_/MeOH 20:1). MS (ESI): m/z =[M+H]^+^ = 573.5, 617.5 (100%), 661.6; [M+Ac]^-^ = 631.4, 675.4 (100%), 719.5. ^1^H-NMR (600 MHz, CDCl_3_): *δ* = 7.33 (d, *J* = 8.3 Hz, 2H), 7.23 (d, *J* = 8.3 Hz, 2H), 4.53 (s, 2H), 3.80-3.51 (m, 34.0H), 2.42 (brs, 1H), 1.73 (s, 2H), 1.35 (s, 6H), 0.70 (s, 9H). ^13^C-NMR (150 MHz, CDCl_3_): *δ* = 149.8, 135.1, 127.5 (2C), 126.3 (2C), 73.2, 72.6, 70.7 (m), 70.4, 69.4, 61.9, 57.0, 38.6, 32.5, 31.9 (3C), 31.7 (2C). IR (cm^-1^): 2957, 2865, 1465, 1350, 1249, 1097, 948, 816, 670, 632. IR (cm-^1^): 2957, 2865, 1465, 1350, 1249, 1097, 948, 816, 670, 632. HRMS (ESI^+^) (m/z): [M+H]^+^ calculated for C_33_H_61_O_10_ (n=9): 617.4265; found: 617.4269.

## Synthesis of *tert*-Butylbenzylalcohol polyethoxylate (**VII**)

*tert*-Butylbenzylalcohol (2.0 g, 11.9 mmol) was dissolved in anhydrous CH_2_Cl_2_ (50 mL) and cooled in an ice/water bath. NEt_3_ (2.1 mL, 14.3 mmol) was added slowly followed by the dropwise addition of MsCl (1.15 mL, 14.3 mmol). The reaction was warmed slowly to ambient temperature and further stirred overnight. An aqueous saturated NaHCO_3_ solution (100 mL) was added as well as EtOAc (150 mL) and the reaction mixture was agitated vigorously. The aqueous phase was extracted with EtOAc (30 mL) and the combined organic phases were washed repeatedly with water (50 mL) and brine (50 mL). After drying and filtration, the organic phase was then concentrated in vacuo to yield 2.13 g (Yield: 74%) of a clear oil, that was engaged directly in the next step.

PEG400 (9.0 g, 22.5 mmol) was dissolved in anhydrous THF (60 mL) under Argon and the mixture was cooled in an ice/water bath. tBuOK (2.53 g, 22.5 mmol) was added portion wise over 2 min and the mixture was stirred 60 min in the cool bath. In the meantime, the Mesylate intermediate (2.11 g, 8.7 mmol) was suspended in THF (8 mL) and the solution was added to the cooled deprotonated PEG400 solution over 4 min. The reaction was warmed slowly to ambient temperature and further stirred overnight. The reaction mixture was filtered over a pad of Celite and the cake was rinsed with CH_2_Cl_2_ (18 mL). The reaction mixture was concentrated, and the residue was dissolved in EtOAc (150 mL) and water (150 mL). The solution was transferred into a separatory funnel and the organic phase was washed successively with water (2 x 100 mL) and brine (50 mL) and finally dried over MgSO_4_ to yield 6.6 g of crude oily residue. This residue was charged on top of a SiO_2_ column and eluted with CH_2_Cl_2_/MeOH (0 to 4%). Concentration of the pure fractions yielded 4.50 g (Yield: 95%) of a pale-yellow oil. R_f_ = 0.32 (CH_2_Cl_2_/MeOH 20:1). ^1^H-NMR (600 MHz, CDCl_3_): *δ* = 7.36 (d, *J* = 8.3 Hz, 2H), 7.27 (d, *J* = 8.3 Hz, 2H), 4.53 (s, 2H), 3.80-3.49 (m, 31H), 2.20 (brs, OH), 1.72 (s, 2H), 1.31 (s, 9H). ^13^C-NMR (150 MHz, CDCl_3_): *δ* = 150.7, 135.3, 127.8 (2C), 125.4 (2C), 73.2, 72.7, 70.7 (m), 70.6, 70.6, 70.4, 69.4, 61.8, 34.7, 31.5 (3C). IR (cm^-1^): 2962, 2863, 1717, 1462, 1349, 1278, 1100, 947, 829, 671, 633. HRMS (ESI^+^) (m/z): [M+Na]^+^ calculated for C_29_H_52_NaO_10_ (n=9): 583.3458; found: 583.3450.

## (4'-methyl-[1,1'-biphenyl]-4-yl) methanol (**XII**)

4-Bromobenzyl alcohol (1.25 g, 6.7 mmol) was dissolved in toluene/EtOH (1:1, 30 mL) and para-tolylboronic acid (1.00 g, 7.35 mmol) was added to the resulting solution. The reaction mixture was degassed and purged with Argon. Pd(PPh_3_)_4_ (370 mg, 0.32 mmol) followed by K_2_CO_3_ (1.85 g, 13.4 mmol) dissolved in water (7 mL) were then added to the flask. After 10 min the reaction vessel was immersed in a preheated oil bath (90°C) and the mixture was stirred for 24 hrs. The reaction mixture was concentrated and toluene (50 mL) was added to the residue. The crude product was concentrated to yield 5.5 g of crude black liquid. This residue was charged on top of a SiO_2_ column and eluted with petroleum ether/EtOAc (0 to 17%). Concentration of the pure fractions yielded 1.25 g (Yield: 94%) of a light yellow crystalline solid. R_f_ = 0.37 (toluene/EtOAc 4:1). m.p. > 250°C. ^1^H-NMR (600 MHz, CDCl_3_): *δ* = 7.58 (d, *J* = 8.2 Hz, 2H), 7.49 (d, *J* = 8.2 Hz, 2H), 7.43 (d, *J* = 8.2 Hz, 2H), 7.26 (d, *J* = 8.2 Hz, 2H), 4.74 (s, 2H), 2.40 (s, 3H), 1.61 (brs, OH). ^13^C-NMR (150 MHz, CDCl_3_): *δ* = 140.7, 139.7, 138.1, 137.3, 129.7 (2C), 127.6 (2C), 127.3 (2C), 127.1 (2C), 65.3, 21.3. IR (cm^-1^): 2915, 2857, 1498, 1047, 1007, 798, 656, 637, 620, 443.HRMS (ESI^+^) (m/z): [M-H_2_O+H]^+^ calculated for C_14_H_13_: 181.1017; found: 181.1024.

## Synthesis of (4'-methyl-[1,1'-biphenyl]-4-yl)methanol polyethoxylate (**XIII**)

Benzyl alcohol **XII** (1.17 g, 5.90 mmol) was dissolved in anhydrous CH_2_Cl_2_ (40 mL) and cooled in an ice/water bath. NEt_3_ (4.3 mL, 30.8 mmol) was added followed by the slow addition of a solution of MsCl (1.45 mL, 18.7 mmol). The reaction warmed slowly to ambient temperature and was further stirred overnight. An aqueous saturated NaHCO_3_ solution (80 mL) as well as CH_2_Cl_2_ (40 mL) were added and the reaction mixture was agitated vigorously. The aqueous phase was back extracted with CH_2_Cl_2_ (60 mL). The combined organic phases were washed successively with water (50 mL) and brine (50 mL) and dried over MgSO_4_. The organic phase was then concentrated in vacuo. Toluene (20 mL) was added to the residue and the crude product was concentrated to yield 1.37 g (Yield: 84%) of an orange yellow solid, that was engaged directly in the next step. R_f_ = 0.70 (petroleum ether/EtOAc 5:1).

PEG400 (3.75 g, 9.41 mmol) was dissolved in anhydrous mTHF (50 mL) under Argon and the mixture was cooled in an ice/water bath. tBuOK (1.05 g, 9.41 mmol) was added portion wise over 1 min and the mixture was stirred 90 min in the cool bath. In the meantime, the Mesylate intermediate (1.30 g, 4.70 mmol) was suspended in mTHF (12 mL) and the solution was added to the cooled deprotonated PEG400 solution over 3 min. The reaction was warmed slowly to ambient temperature and further stirred 12 days. The reaction mixture was filtered over a pad of Celite and the cake was rinsed with CH_2_Cl_2_ (150 mL). The reaction mixture was concentrated and EtOAc (180 mL) as well as water (50 mL) were added. The solution was transferred into a separatory funnel and extracted vigorously. After phase separation the organic phase was washed successively with water/brine (1:1; 5 x 50 mL) and finally dried over MgSO_4_ to yield 1.8 g of crude oily residue. The residue was dissolved in MeOH (80 mL) and an aqueous solution of NaOH 2 M (80 mL) was added slowly. The reaction mixture was stirred 45 min at ambient temperature, before aqueous HCl 1 M (120 mL) was added. The volatiles were removed under light vacuum. The aqueous residue was extracted with CH_2_Cl_2_ (3 x 100 mL). The combined organic phases were washed with brine (30 mL) and dried over MgSO_4_ to yield 1.4 g of crude oily residue. This residue was charged on top of a SiO_2_ column and eluted with CH_2_Cl_2_/MeOH (0 to 6%). Concentration of the pure fractions yielded 1.16 g (Yield: 42,6%) of a dark brown clear oil that gives a semi solid while stored at 2-8°C. R_f_ = 0.71-0.47 (CH_2_Cl_2_/MeOH 10:1). ^1^H-NMR (600 MHz, CDCl_3_): *δ* = 7.55 (d, *J* = 8.3 Hz, 2H), 7.48 (d, *J* = 8.2 Hz, 2H), 7.39 (d, *J* = 8.3 Hz, 2H), 7.24 (d, *J* = 8.2 Hz, 2H), 4.60 (s, 2H), 3.73-3.59 (m, 38H), 2.39 (s, 3H), 2.28 (brs, OH). ^13^C-NMR (150 MHz, CDCl_3_): *δ* = 140.6, 138.2, 137.2 (2C), 129.6 (2C), 128.3 (2C), 127.1 (4C), 73.2, 72.7, 70.7 (m), 70.5, 69.6, 61.9, 21.2. IR (cm^-1^): 2864, 1501, 1455, 1349, 1250, 1095, 948, 806, 671, 565. HRMS (ESI^+^) (m/z): [M+Na]^+^ calculated for C_23_H_50_NaO_10_ (n=9): 617.3302; found: 617.3320.

## Synthesis of polyethoxylated (4-butylphenyl)methanol (**XIV**)

4-Butylbenzyl alcohol (1.00 g, 6.09 mmol) was dissolved in anhydrous CH_2_Cl_2_ (40 mL) and cooled in an ice/water bath. NEt_3_ (4.5 mL, 32.5 mmol) was added followed by the slow addition of a solution of MsCl (1.5 mL, 19.4 mmol). The reaction warmed slowly to ambient temperature and was further stirred overnight. An aqueous saturated NaHCO_3_ solution (80 mL) as well as CH_2_Cl_2_ (100 mL) were added and the reaction mixture was agitated vigorously. The aqueous phase was back extracted with CH_2_Cl_2_ (80 mL). The combined organic phases were washed successively with water (50 mL) and brine (50 mL) and dried over MgSO_4_. The organic phase was then concentrated in vacuo. Toluene (20 mL) was added to the residue and the crude product was concentrated to yield 1.2 g (Yield: 81%) of an orange oil, that was engaged directly in the next step. R_f_ = 0.93 (petroleum ether/EtOAc 5:1).

PEG400 (3.6 g, 9.08 mmol) was dissolved in anhydrous mTHF (50 mL) under Argon and the mixture was cooled in an ice/water bath. tBuOK (1.0 g, 9.08 mmol) was added portion wise over 2 min and the mixture was stirred 90 min in the cool bath. In the meantime, the Mesylate intermediate (1.1 g, 4.54 mmol) was suspended in mTHF (15 mL) and the solution was added to the cooled deprotonated PEG400 solution over 3 min. The reaction was warmed slowly to ambient temperature and further stirred 12 days. The reaction mixture was filtered over a pad of Celite and the cake was rinsed with CH_2_Cl_2_ (50 mL). The reaction mixture was concentrated and EtOAc (180 mL) as well as water (50 mL) were added. The solution was transferred into a separatory funnel and extracted vigorously. After phase separation the organic phase was washed successively with water/brine (1:1; 5 x 50 mL) and finally dried over MgSO_4_ to yield 1.3 g of dark yellow oily residue. The residue was dissolved in MeOH (80 mL) and an aqueous solution of NaOH 2 M (80 mL) was added slowly. The reaction mixture was stirred 45 min at ambient temperature, before aqueous HCl 1 M (120 mL) was added. The volatiles were removed under light vacuum. The aqueous residue was extracted with CH_2_Cl_2_ (3 x 100 mL). The combined organic phases were washed with brine (30 mL) and dried over MgSO_4_ to yield 1.3 g of crude oily residue. This residue was charged on top of a SiO_2_ column and eluted with CH_2_Cl_2_/MeOH (0 to 6%). Concentration of the pure fractions yielded 750 mg (Yield: 30%) of a yellowish clear oil. R_f_ = 0.77-0.49 (CH_2_Cl_2_/MeOH 10:1). ^1^H-NMR (600 MHz, CDCl_3_): *δ* = 7.24 (d, *J* = 8.0 Hz, 2H), 7.14 (d, *J* = 8.0 Hz, 2H), 4.52 (s, 2H), 3.73-3.59 (m, 33H), 2.59 (t, 2H, *J* = 7.7 Hz ), 2.43 (brs, OH), 1.58 (m, 2H), 1.34 (m, 2H), 0.92 (t, 3H, *J* = 7.4 Hz ). ^13^C-NMR (150 MHz, CDCl_3_): *δ* = 142.5, 135.6, 128.5 (2C), 128.0 (2C), 73.3, 72.7, 70.7 (m), 70.5, 69.5, 61.9, 35.5, 33.8, 22.5, 14.1. IR (cm^-1^): 2953, 2929, 2862, 1458, 1351, 1248, 1095, 946, 848, 650, 667, 614, 489. HRMS (ESI^+^) (m/z): [M+Na]^+^ calculated for C_29_H_52_NaO_10_ (n=9): 583.3458; found: 583.3478.

## Synthesis of 1-methyl-4-(2,4,4-trimethylpentan-2-yl)benzene (**XV**)

At ambient temperature and under stirring, the reaction vessel was charged with toluene (V = 600 mL, 4 vol.) and nonafluoro-1-butanesulfonic acid (V=4.8 mL) was added in one portion. The solution was cooled to 0°C and a mixture of diisobutylene (V= 150 mL) premixed in toluene (V= 300 mL) was added slowly over 40-100 min to the reaction mixture using a dropping funnel, while cooling the reaction mixture in the ice/water bath. Then the reaction mixture was stirred 120 min at ambient temperature before aq. saturated NaHCO_3_ (V= 300 mL) was added in one portion to quench the reaction. The reaction mixture was stirred for 60 min and the content of the flask was transferred to a separatory funnel. The aqueous phase was removed after phase separation and the organic phase was washed with water (2 x V = 500 mL). The organic phase was concentrated thoroughly in vacuo to yield the crude product as a light brown oily residue (195.5 g, quantitative yield). R_f_ = 0.65 (Petrol ether 40-60 100%). ^1^H-NMR (600 MHz, CDCl_3_): δ = 7.28 (d, *J* = 8.2 Hz, 2H), 7.10 (d, *J* = 8.2 Hz, 2H), 2.34 (s, 3H), 1.75 (s, 2H), 1.38 (s, 6H), 0.75 (s, 9H). ^13^C-NMR (150 MHz, CDCl_3_): δ = 147.3, 134.7, 128.6 (2C), 126.1 (2C), 57.1, 38.4, 32.5, 31.9 (3C), 31.7 (2C), 21.0. IR (cm^-1^): 2953, 2906, 2867, 1513, 1469, 1364, 1251, 1020, 815, 649, 489.

## Synthesis of 1-(bromomethyl)-4-(2,4,4-trimethylpentan-2-yl)benzene (**XVI**)

para-Substituted toluene **XV** (195 g, 954 mmol) was dissolved in cyclohexane (1560 mL, 8 vol.) and NBS (170 g, 954 mmol) and AIBN (0.8 g, 4.8 mmol) are added while stirring. The mixture was heated to 70°C until completion and cooled down to ambient temperature. The solid was filtered off and the cake was rinsed with cHex (100 mL). The filtrate was transferred into a separatory funnel and the organic phase was washed with water (2 x 500 mL). The organic phase was concentrated in vacuo to yield a crude residue as a brown clear oil. IPA (2 mL/g of residue) was added to the crude and the solution cooled to -20°C. After 1 hr at -20°C a few seed crystals (10-50 mg) were added. The flask was left at -20°C overnight. The solid was filtered off, rinsed with cold IPA (50 mL) to yield an off-white solid and the corresponding mother liquor. This solid was re-dissolved in IPA (2 mL/g solid) and water (0.2 mL/g solid), using the water bath of the rotavapor (45°C, 5 min). The solution was cooled to +2-8°C. After 1 hr at +2-8°C a few seed crystals (10-50 mg) were added and the flask was left at +2-8°C overnight and further cooled to -20°C for 4 hours. Repeat crystallization if necessary. The precipitate was filtered off, rinsed with cold IPA (50 mL) to yield colorless needles. The solid was dried in the vacuum oven (30°C, <15 mbar, 24 hrs followed by 20°C, <15 mbar, 24 hrs) to yield 50-80 g (20-30% yield over 2 steps). R_f_ = 0.43 (Petrol ether 40-60 100%). m.p. (DSC) = 53°C. ^1^H-NMR (600 MHz, CDCl_3_): δ = 7.34 (d, *J* = 8.4 Hz, 2H), 7.30 (d, *J* = 8.4 Hz, 2H), 4.50 (s, 2H), 1.73 (s, 2H), 1.36 (s, 6H), 0.72 (s, 9H). ^13^C-NMR (150 MHz, CDCl_3_): δ = 150.9, 134.8, 128.7 (2C), 126.7 (2C), 57.0, 38.7, 33.9, 32.5, 31.9 (3C), 31.6 (2C). IR (cm^-1^): 2953, 2899, 2868, 1511, 1470, 1366, 1252, 1230, 1204, 1095, 830, 648, 628, 606, 573, 454.

## Synthesis of the side product **XVII**

para-Substituted benzyl bromide **XVI** (502 mg, 1.77 mmol) was dissolved in cyclohexane (6 mL). NBS (474 mg, 417 mmol) was added as well as AIBN (16 mg, 0.09 mmol) while stirring (400 rpm). The mixture was heated to 80°C for 4 hours. The reaction mixture was cooled to ambient temperature and 20 mL cyclohexane was added. The content of the flask was transferred into a separatory funnel and the organic phase was washed with water (2 x 5 mL) followed by brine (5 mL). The solvent was removed under vacuo to yield a yellowish residue (720 mg). This residue was charged on top of a SiO_2_ column and eluted with hexane (100%). Concentration of the pure fractions yielded 358 mg (Yield: 56%) of a clear colorless viscous oil. R_f_ = 0.47 (hexane). ^1^H-NMR (600 MHz, CDCl_3_): δ = 7.47 (d, *J* = 8.4 Hz, 2H), 7.36 (d, *J* = 8.4 Hz, 2H), 6.65 (s, 1H), 1.74 (s, 2H), 1.36 (s, 6H), 0.72 (s, 9H). ^13^C-NMR (150 MHz, CDCl_3_): δ = 152.6, 139.1, 126.5 (2C), 126.1 (2C), 57.0, 41.3, 38.9, 32.5, 31.9 (3C), 31.5 (2C). IR (cm^-1^): 2955, 2898, 2871, 1607, 1469, 1411, 1365, 1251, 1143, 1095, 1017, 836, 746, 667.

## Synthesis of **VI** from **XVI**

A reaction flask was filled with PEG400 (700 g, 1.77 mol) and heated to 60°C before tBuOK (47.5 g, 423 mmol) was added portion wise under stirring. After addition, the reaction mixture was heated 30 min at 60°C. The solid crystalline benzyl bromide intermediate **XVI** (100 g) was added in one portion to the deprotonated PEG. After 30 min, the reaction mixture was allowed to cool down to ambient temperature and water (1.5 L) was added. Using 1 M HCl aq. solution, the pH was set to 6-7. Optionally, if the final product needs to be almost colorless: Sodium hypochlorite (5% active Cl solution, 10-15 mL) was added dropwise to the reaction vessel. The content of the vessel was transferred to a separatory funnel. Water (0.5 L) and EtOAc (2 L) were added to the funnel and the phases were shaken vigorously. After phase separation the aqueous phase was removed. Water/brine (20:1) (1 L) was added and the phases were shaken vigorously, after phase separation the aqueous phase was removed. Repeat 2 times. The EtOAc phase was then concentrated under reduced pressure to yield about 200 g of a yellowish clear product. The residue was taken into EtOH (2 L) and transferred to the separatory funnel. cHex (3 L) was added as well as water (100 mL). The phases were shaken vigorously, and the cHex phase was removed. Fresh cHex (1 L) was added, the phases were shaken vigorously, and the cHex phase was removed. The EtOH phase was concentrated under reduced pressure and the product was further dried overnight to yield about 160-170 g (75-80% yield) of a light yellowish clear product. MS (ESI): m/z =[M+H]^+^ = 573.5, 617.5 (100%), 661.6; [M+Ac]^-^ = 631.4, 675.4 (100%), 719.5. ^1^H-NMR (600 MHz, CDCl_3_): *δ* = 7.33 (d, *J* = 8.3 Hz, 2H), 7.23 (d, *J* = 8.3 Hz, 2H), 4.52 (s, 2H), 3.72-3.58 (m, 33H), 2.54 (brs, 1H), 1.72 (s, 2H), 1.34 (s, 6H), 0.70 (s, 9H). ^13^C-NMR (150 MHz, CDCl_3_): *δ* = 149.7, 135.1, 127.4 (2C), 126.2 (2C), 73.2, 72.6, 70.7 (m), 70.5, 69.4, 61.9, 57.0, 38.6, 32.5, 31.9 (3C), 31.6 (2C). IR (cm^-1^): 2957, 2865, 1465, 1350, 1249, 1097, 948, 816, 670, 632. IR (cm-^1^): 2957, 2865, 1465, 1350, 1249, 1097, 948, 816, 670, 632. HRMS (ESI^+^) (m/z): [M+H]^+^ calculated for C_33_H_61_O_10_ (n=9): 617.4265; found: 617.4269.

## Synthesis of the side product bi-functional product **XVIII**

PEG400 (5.01 g, 12.5 mmol) was dissolved in THF (20 mL) at ambient temperature. tBuOK (3.25 g, 28.8 mmol) was added in one portion and the mixture was stirred 30 min. The benzyl bromide **XVI** was added to the deprotonated PEG400 solution at ambient temperature in one portion and the reaction was stirred overnight at ambient temperature. Water (120 g) and HCl (1 M, 10 mL) were added to the reaction mixture. The solution was transferred into a separatory funnel and EtOAc (120 mL) was added and extracted vigorously. After phase separation the organic phase was washed successively with water (2 x 100 mL) and finally concentrated after drying on MgSO_4_ to yield 10.8 g of crude yellowish oily residue. This residue was charged on top of a SiO_2_ column and eluted with CH_2_Cl_2_/MeOH (0 to 15%). Concentration of the pure fractions yielded 9.13 g (Yield: 91%) of a clear light yellowish oil. R_f_ = 0.21-0.78 (CH_2_Cl_2_/MeOH 20:1). ^1^H-NMR (600 MHz, CDCl_3_): δ = 7.33 (d, *J* = 8.2 Hz, 4H), 7.23 (d, *J* = 8.2 Hz, 4H), 4.53 (s, 4H), 3.80-3.49 (m, 36H), 1.73 (s, 4H), 1.35 (s, 12H), 0.71 (s, 18H). ^13^C-NMR (150 MHz, CDCl_3_): δ = 149.8 (2C), 135.2 (2C), 127.5 (4C), 126.3 (4C), 73.2, 70.8, 70.8 (m), 69.4, 57.1 (2C), 38.6 (2C), 32.5 (2C), 31.9 (6C), 31.7 (4C). IR (cm^-1^): 2951, 2861, 1465, 1363, 1249, 1099, 817, 670, 633. HRMS (ESI^+^) (m/z): [M+Na]^+^ calculated for C_48_H_82_NaO_10_ (n=9): 841.5806; found: 841.5797.

# NMR Spectra

## ^1^H-NMR spectra of compound **III**


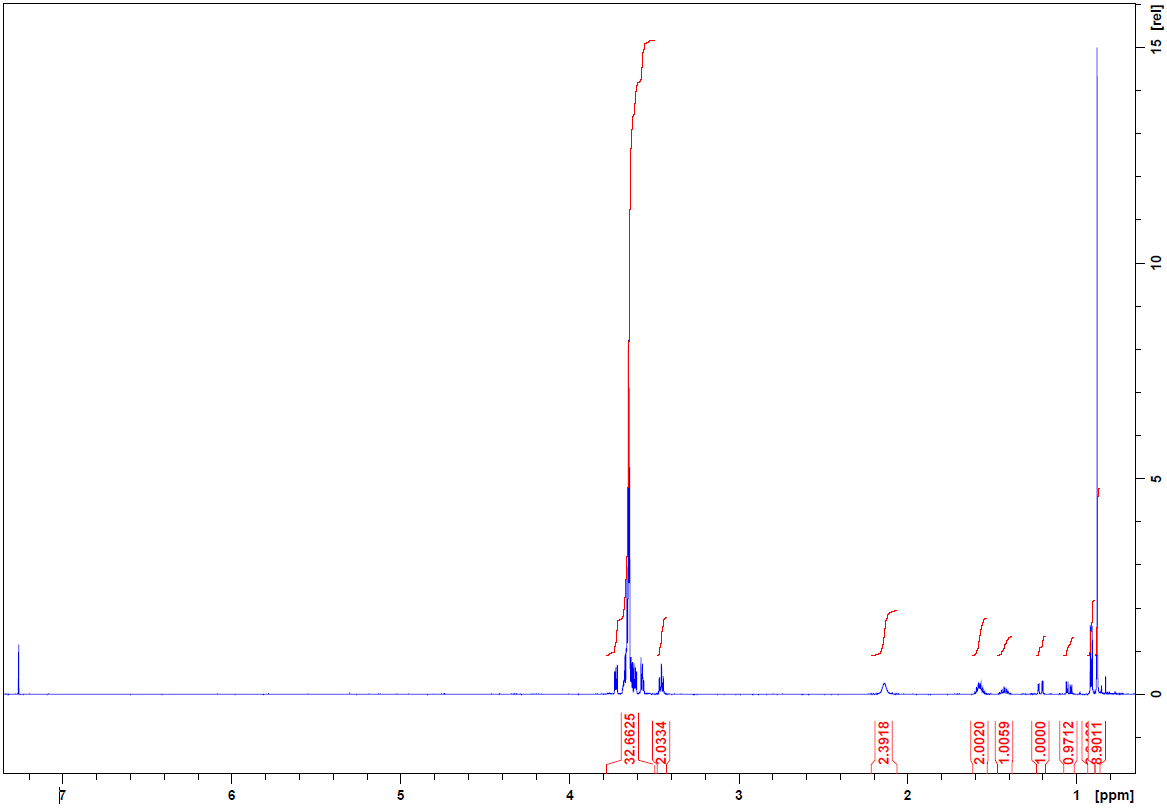

## ^13^C-NMR-Jmod spectra of compound **III**


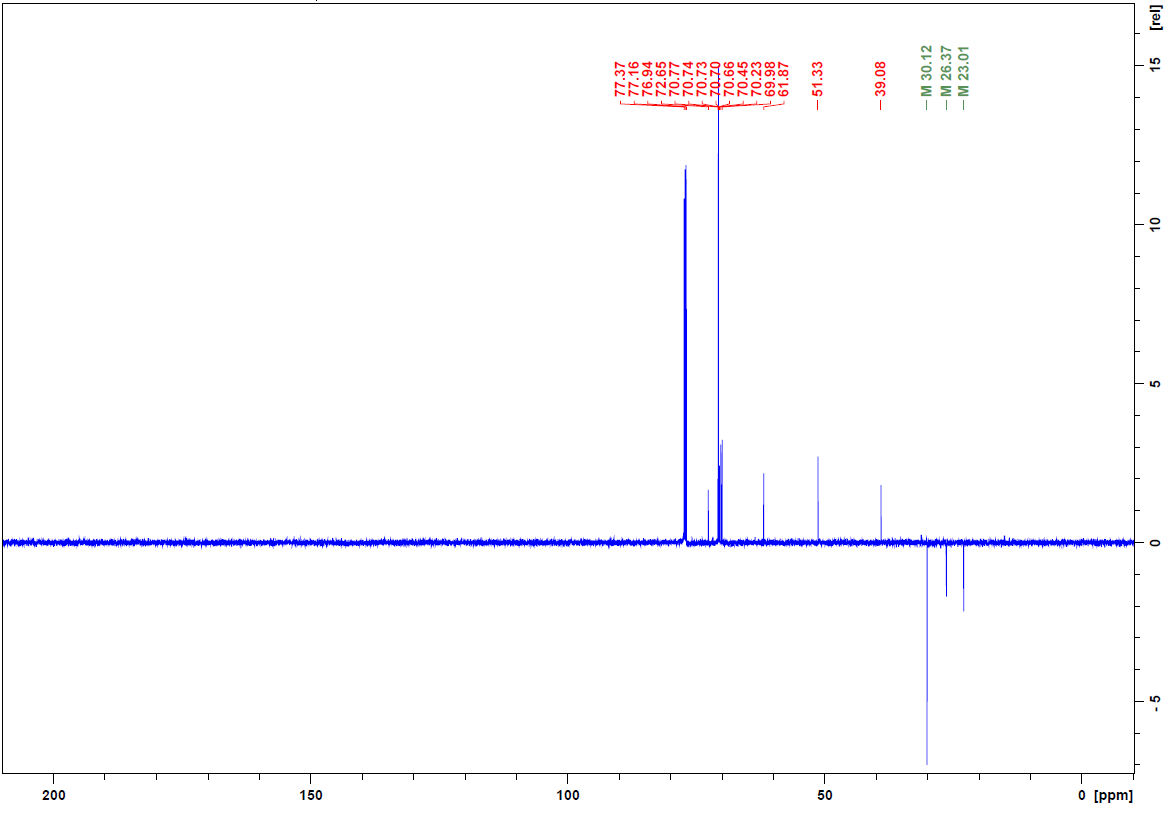


^1^H-NMR spectra of compound **IV**
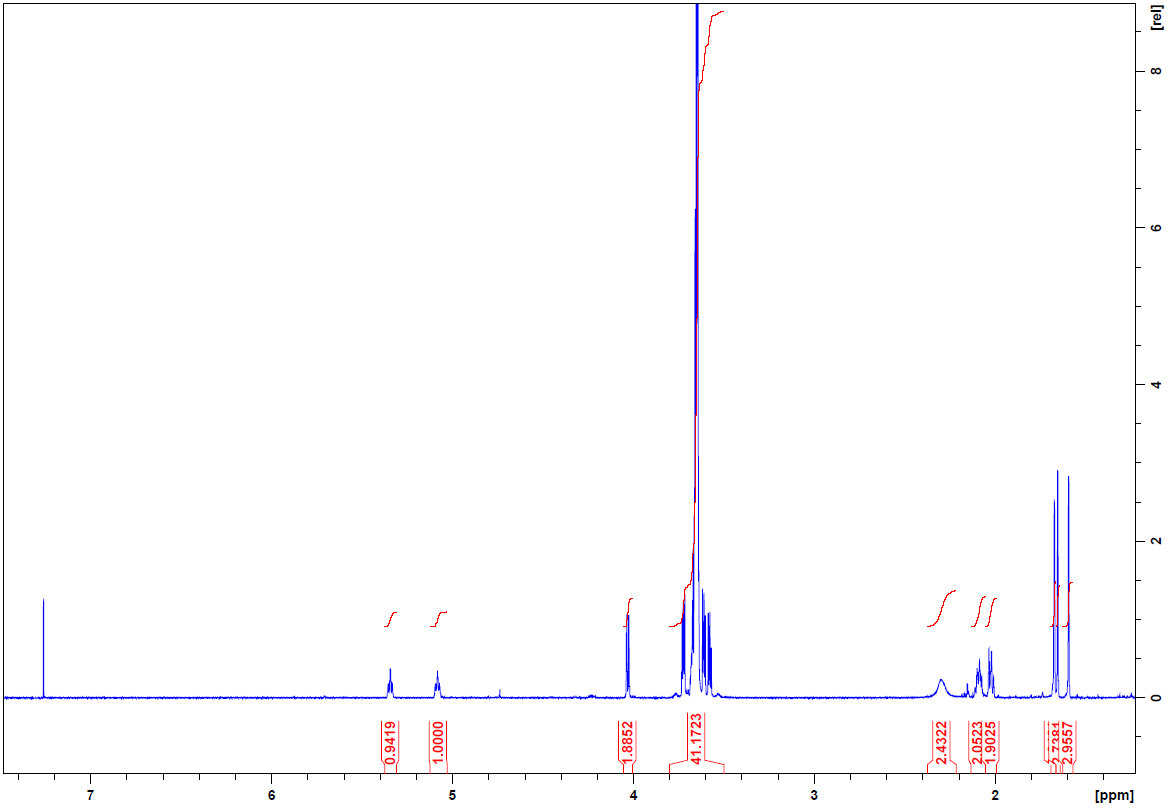

## ^13^C-NMR-Jmod spectra of compound **IV**


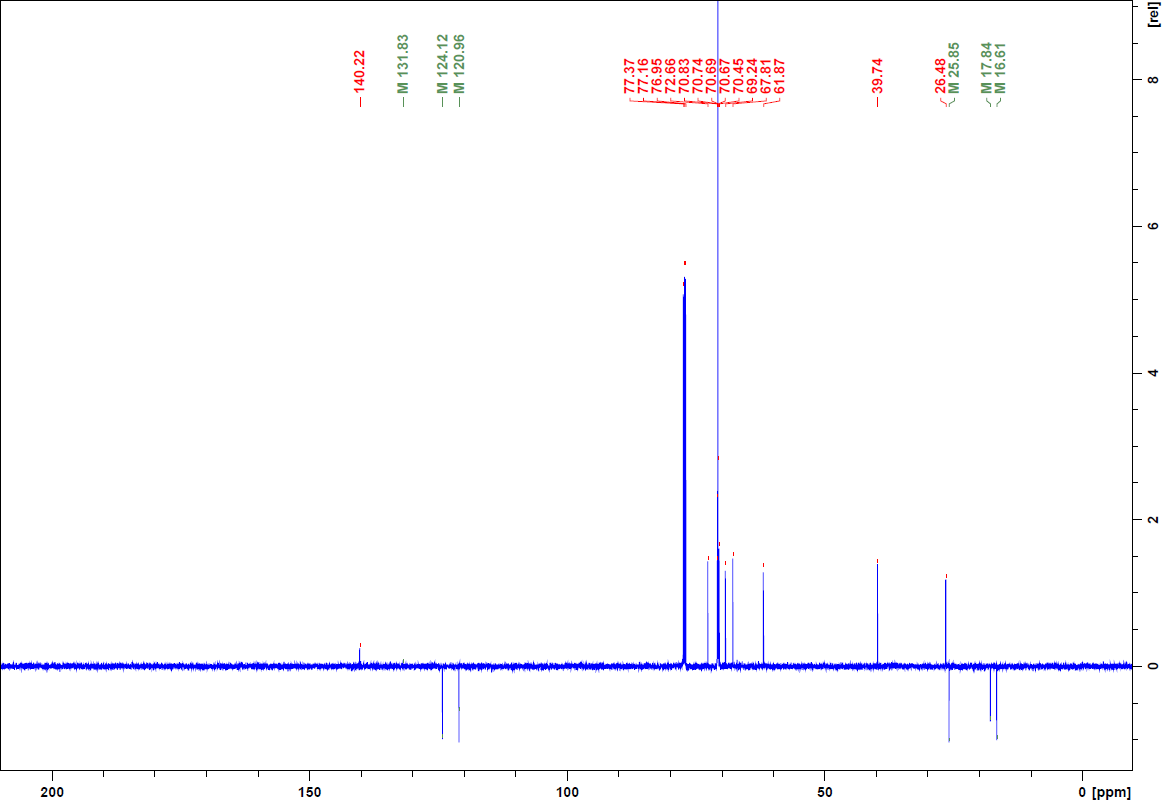


## ^1^H-NMR spectra of compound **V**


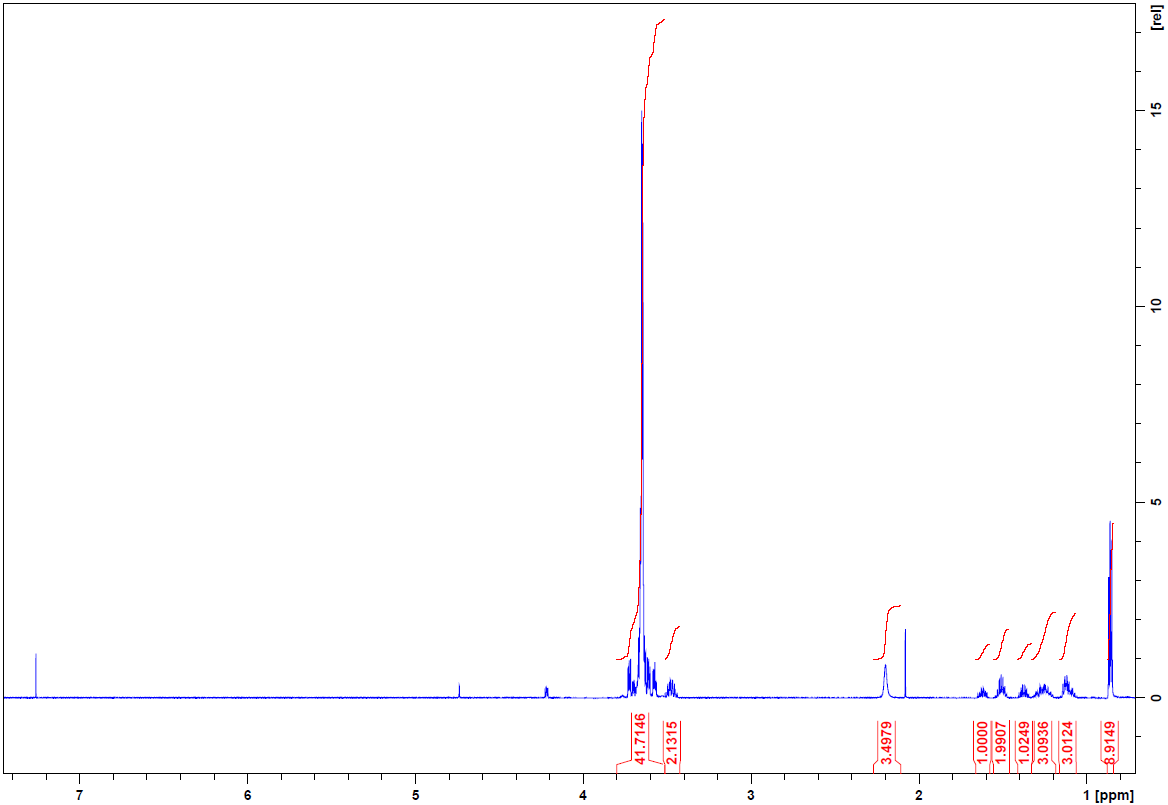

## ^13^C-NMR-Jmod spectra of compound **V**


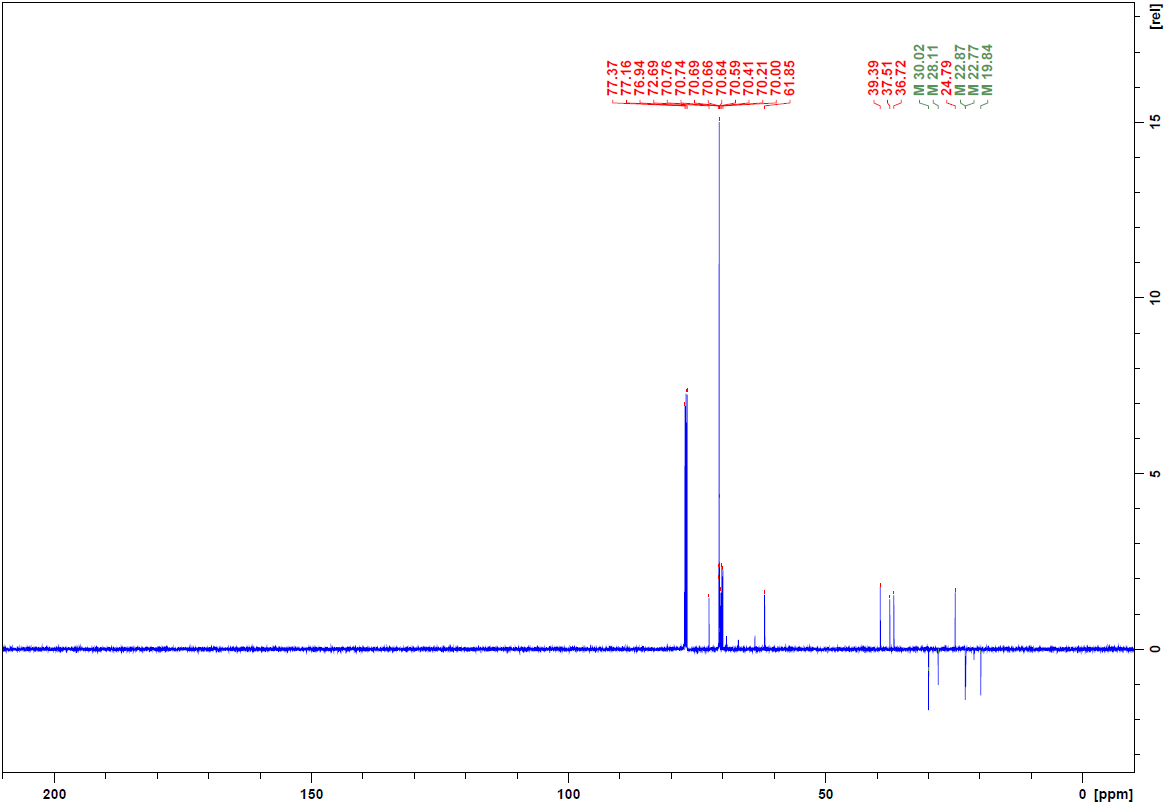


## ^1^H-NMR spectra of compound **VIII**


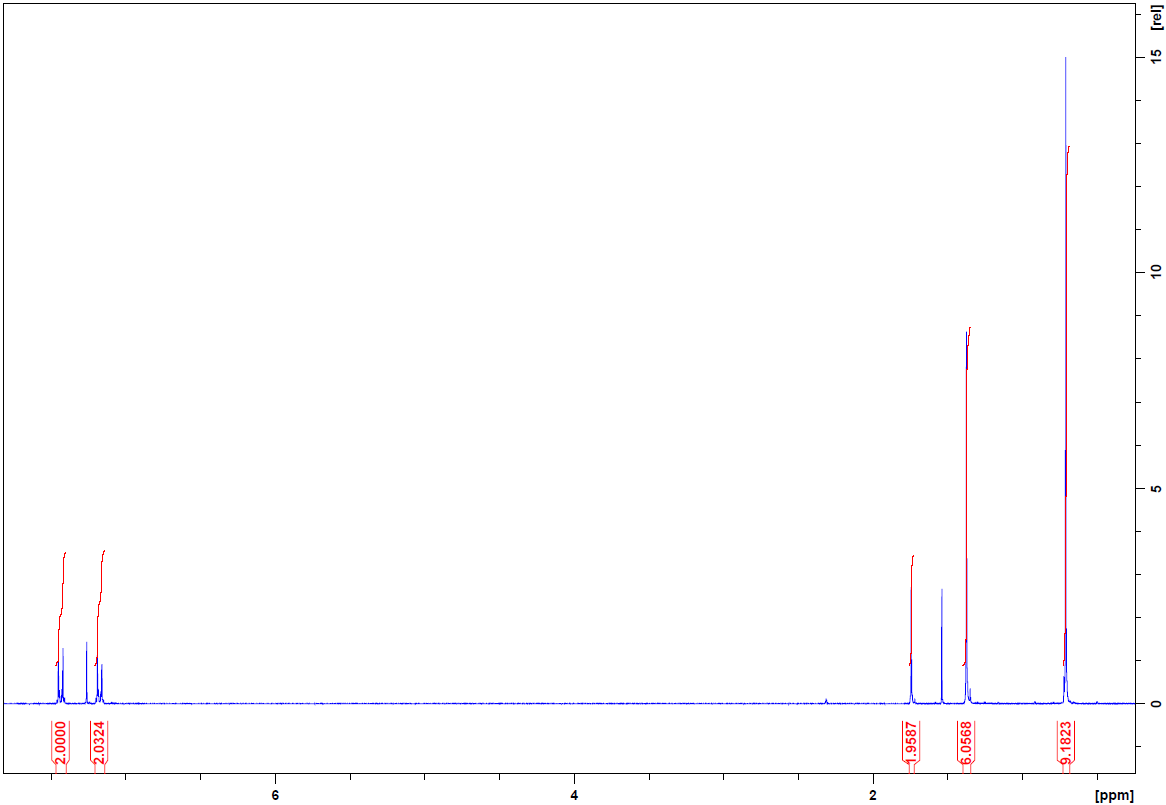

## ^13^C-NMR-Jmod spectra of compound **VIII**


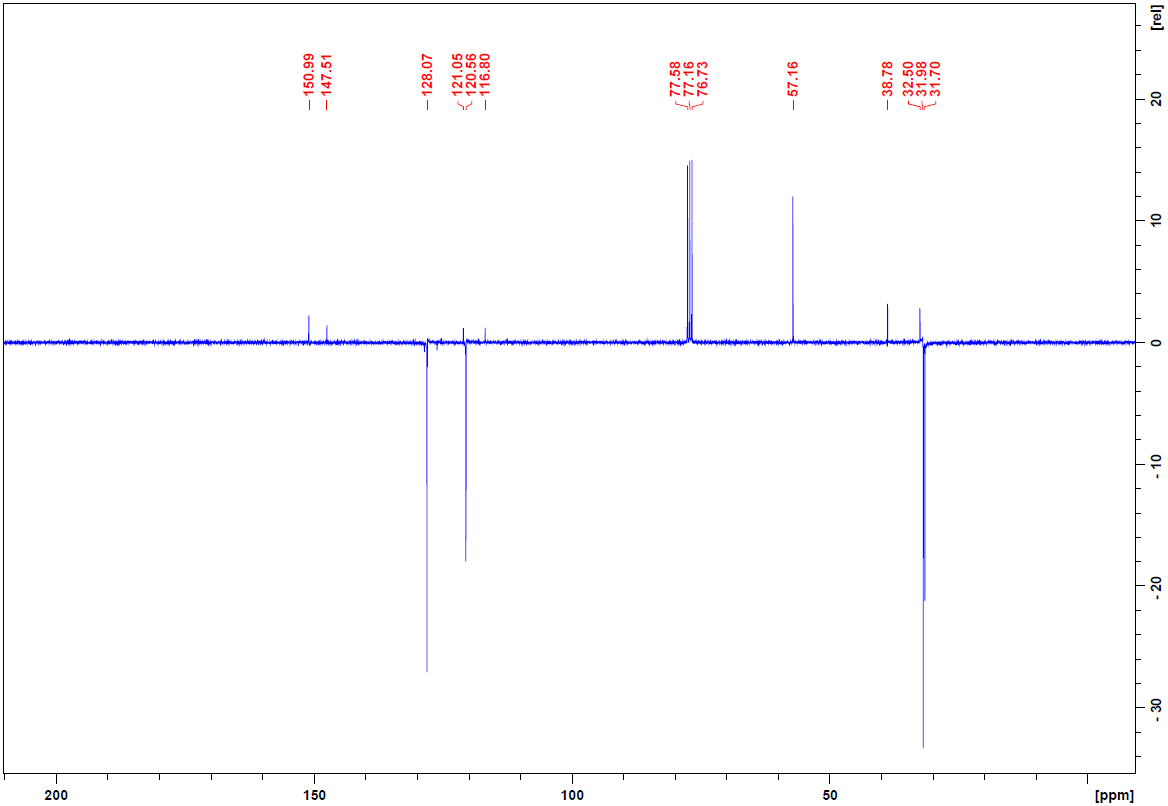


## ^1^H-NMR spectra of compound **IX**


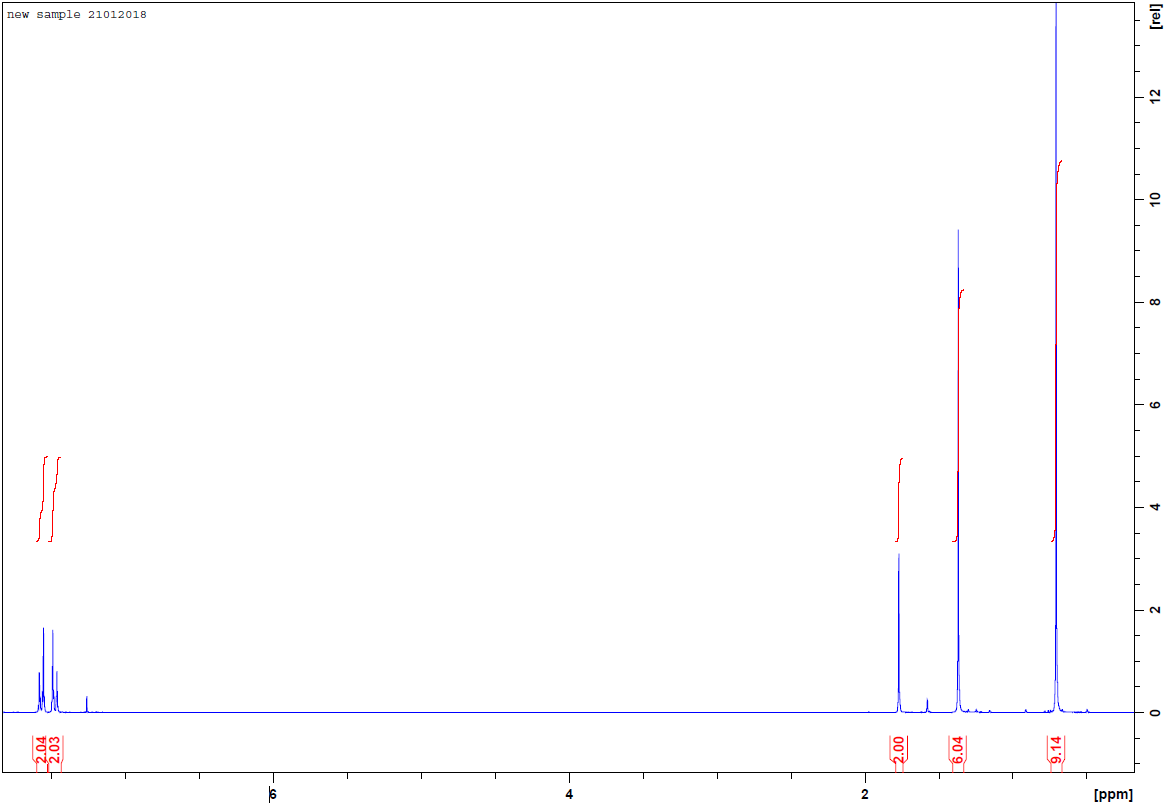

## ^13^C-NMR-Jmod spectra of compound **IX**


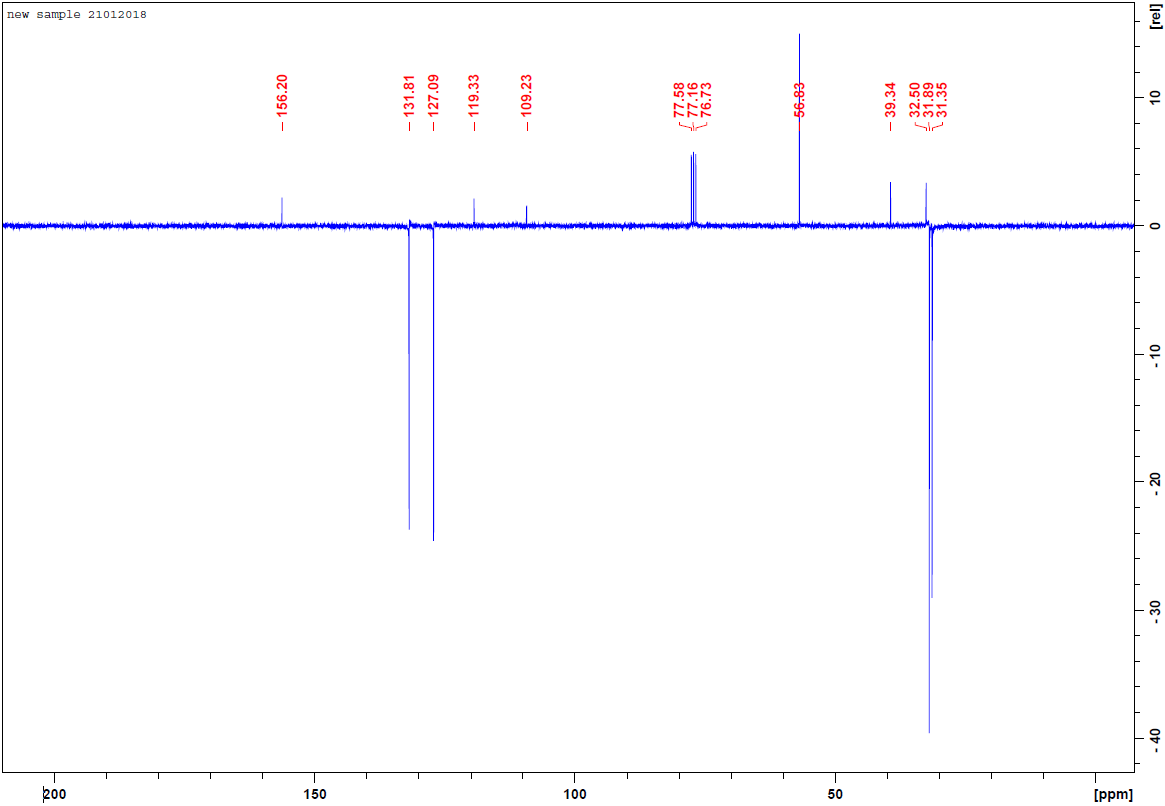


## ^1^H-NMR spectra of compound **X**


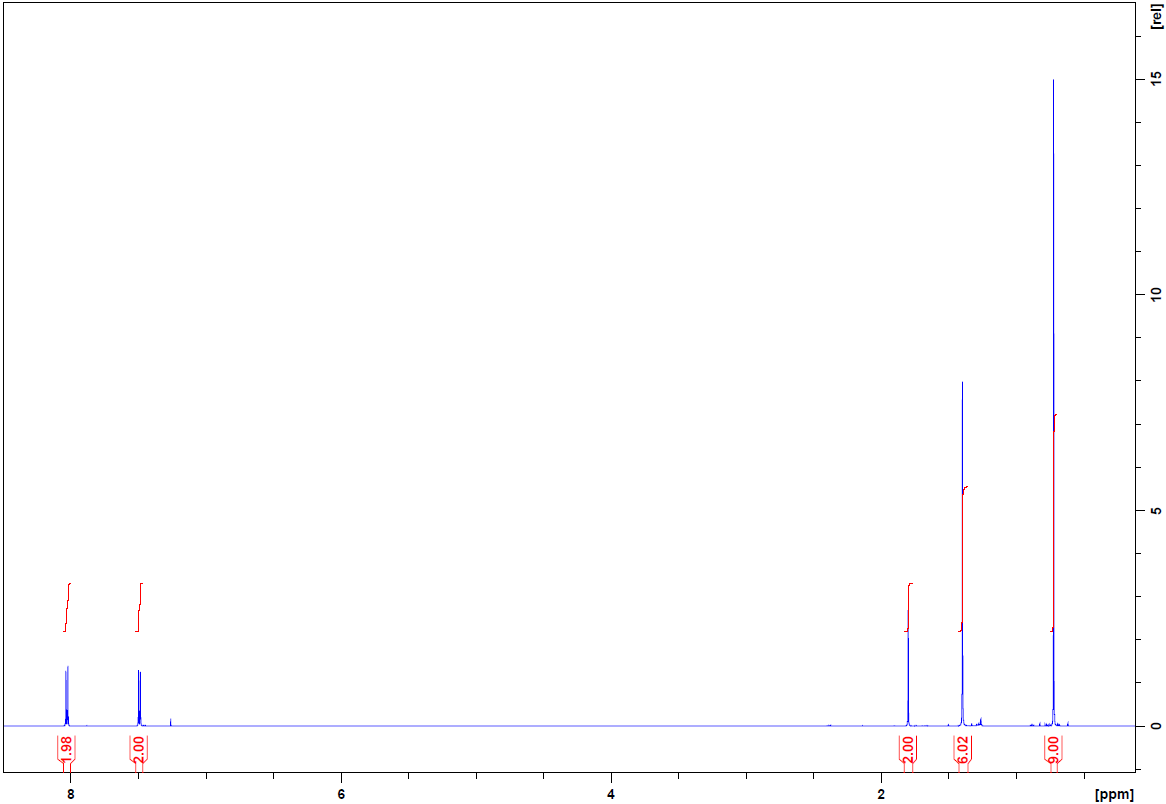

## ^13^C-NMR-Jmod spectra of compound **X**


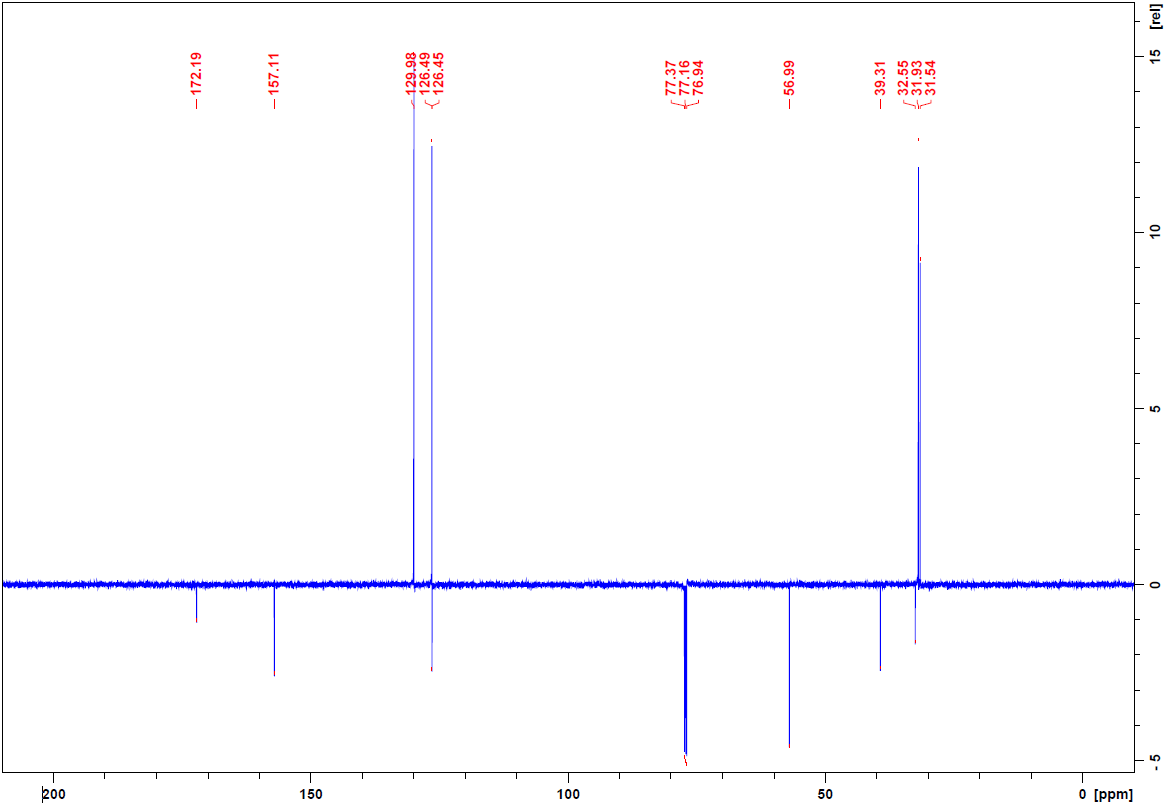


## ^1^H-NMR spectra of compound **XI**


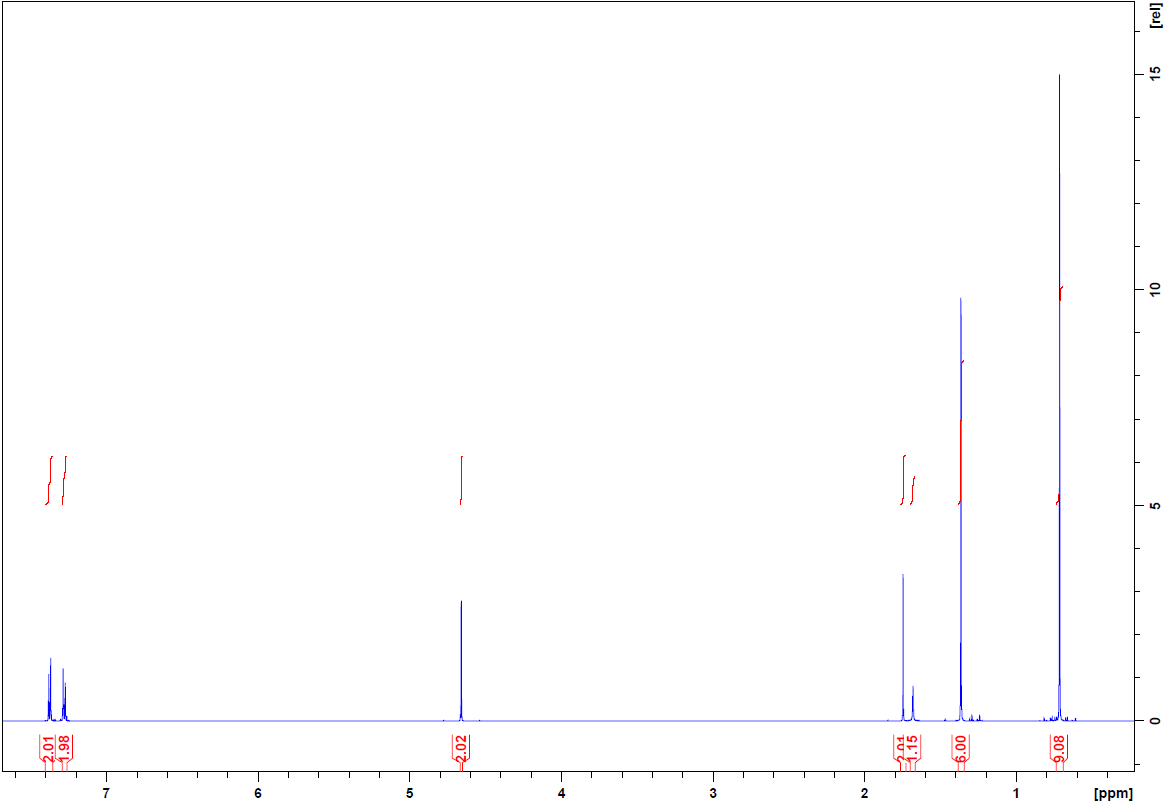

## ^13^C-NMR-Jmod spectra of compound **XI**


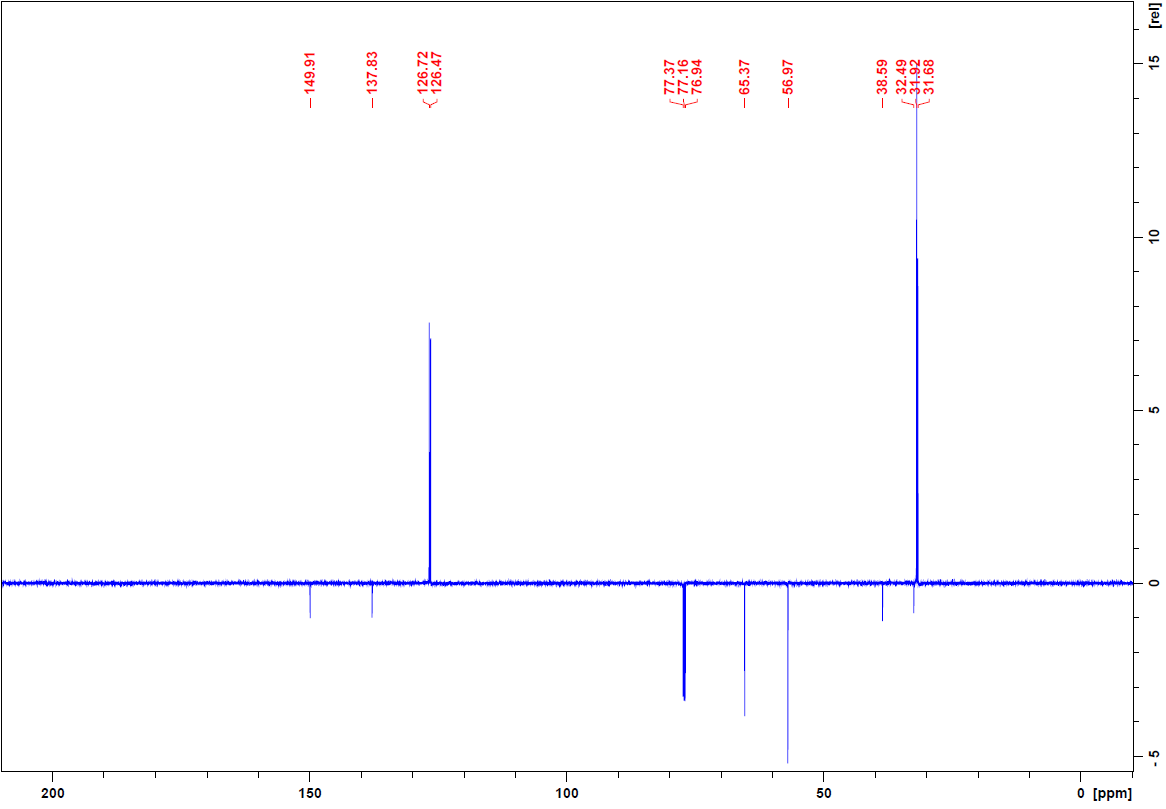


## ^1^H-NMR spectra of compound **VI**


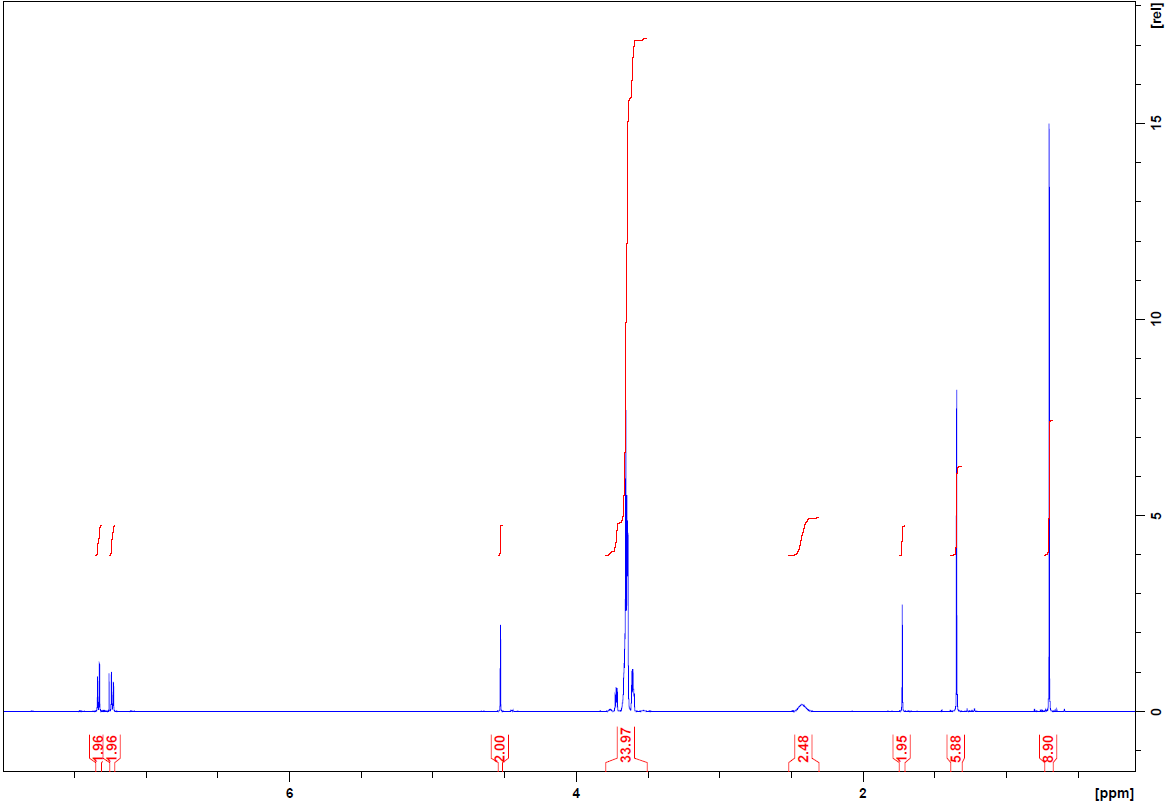

## ^13^C-NMR-Jmod spectra of compound **VI**


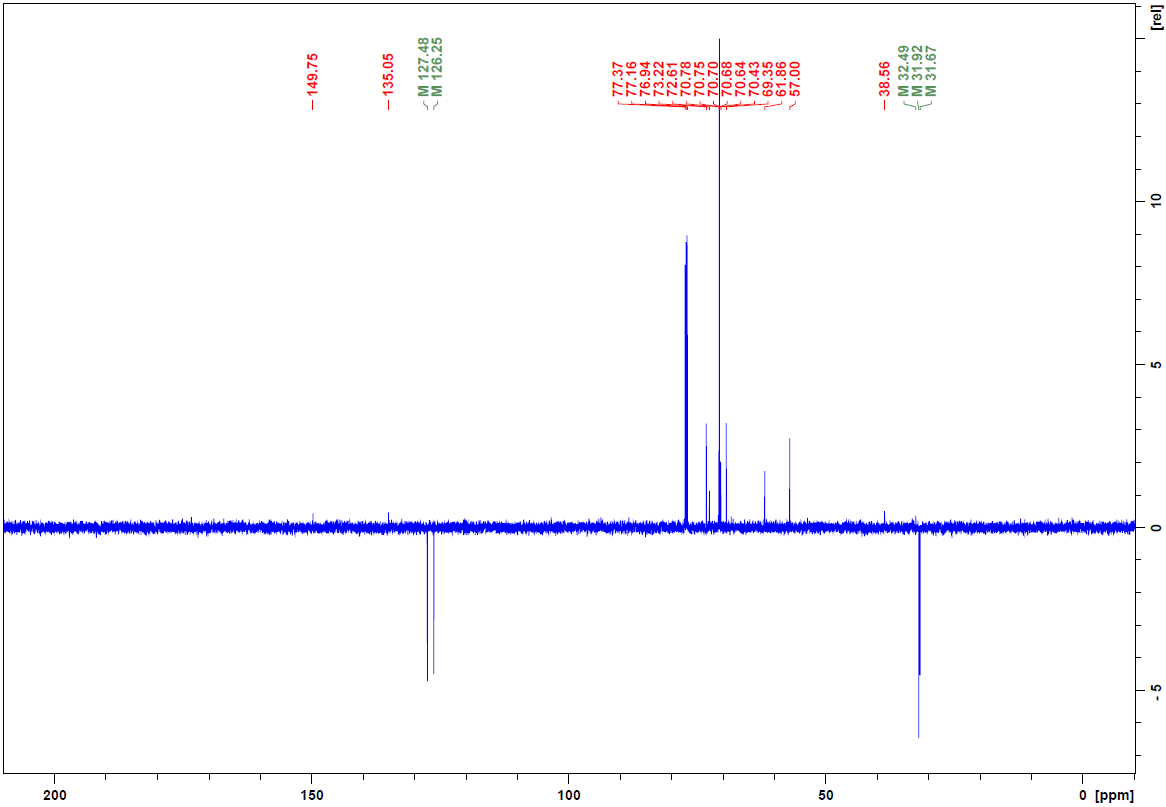


## ^1^H-NMR spectra of compound **VII**


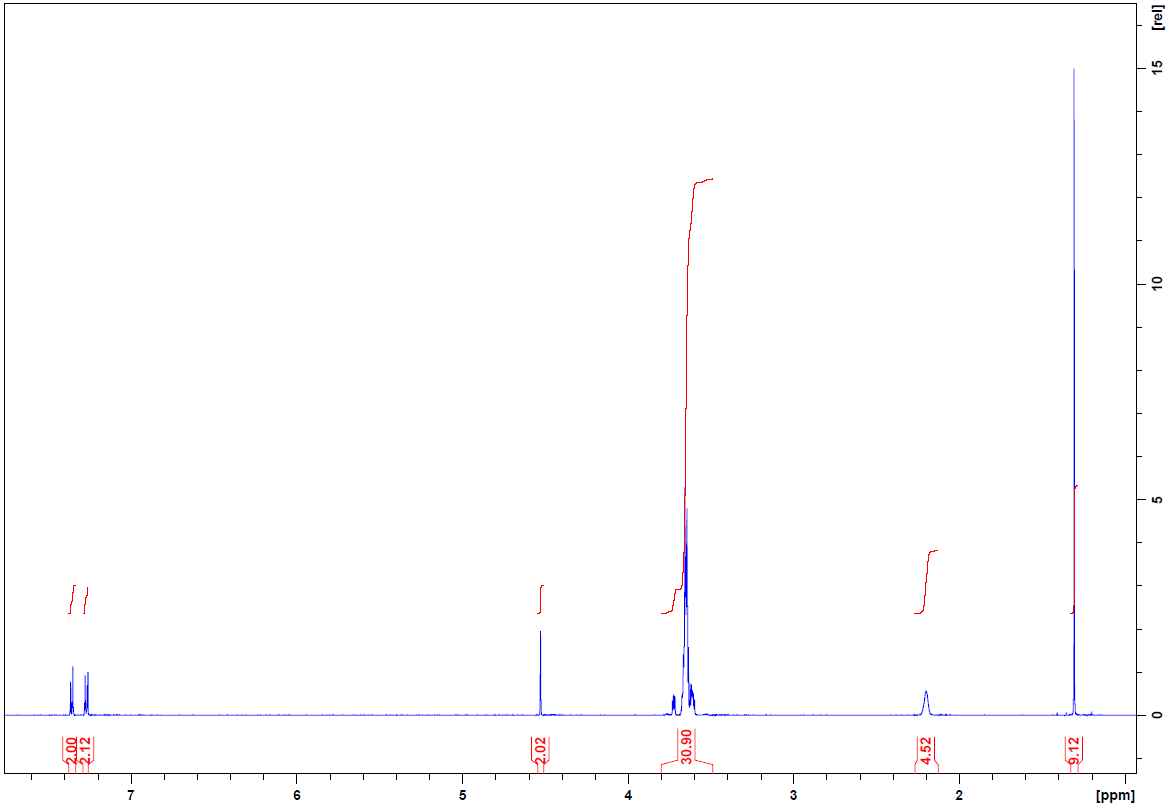

## ^13^C-NMR-Jmod spectra of compound **VII**


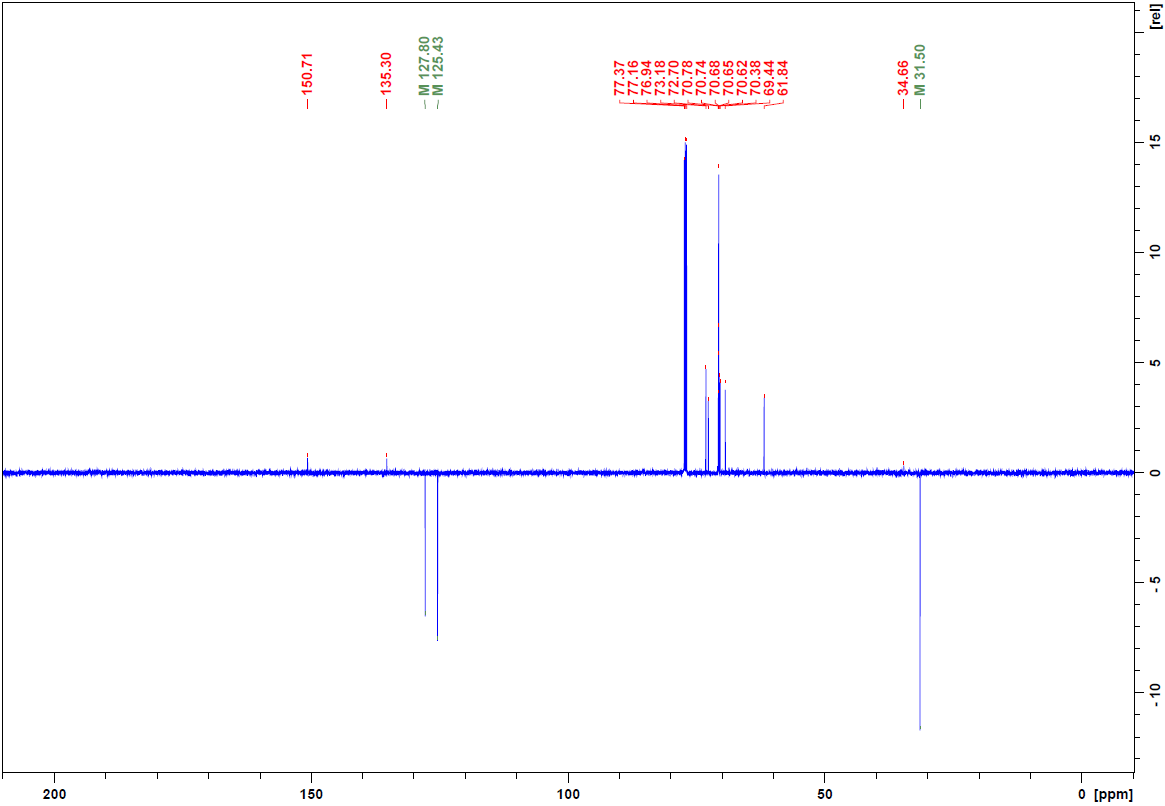


## ^1^H-NMR spectra of compound **XII**


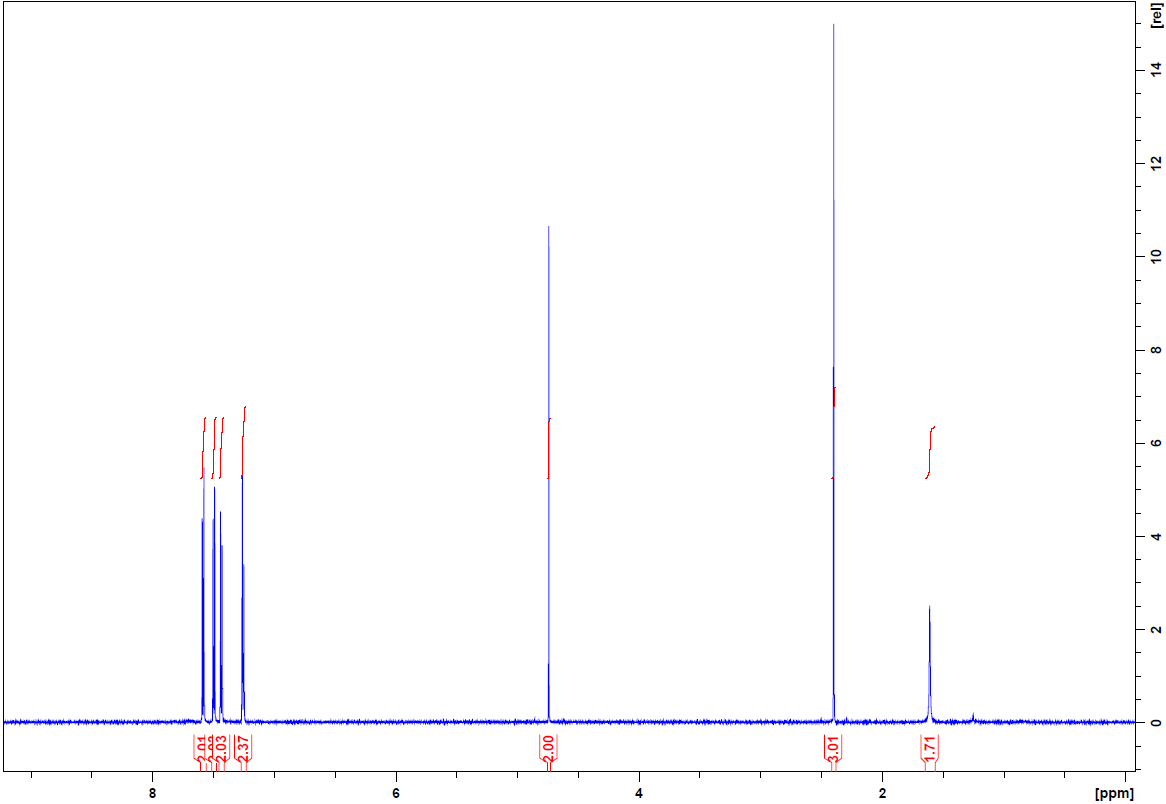

## ^13^C-NMR-Jmod spectra of compound **XII**


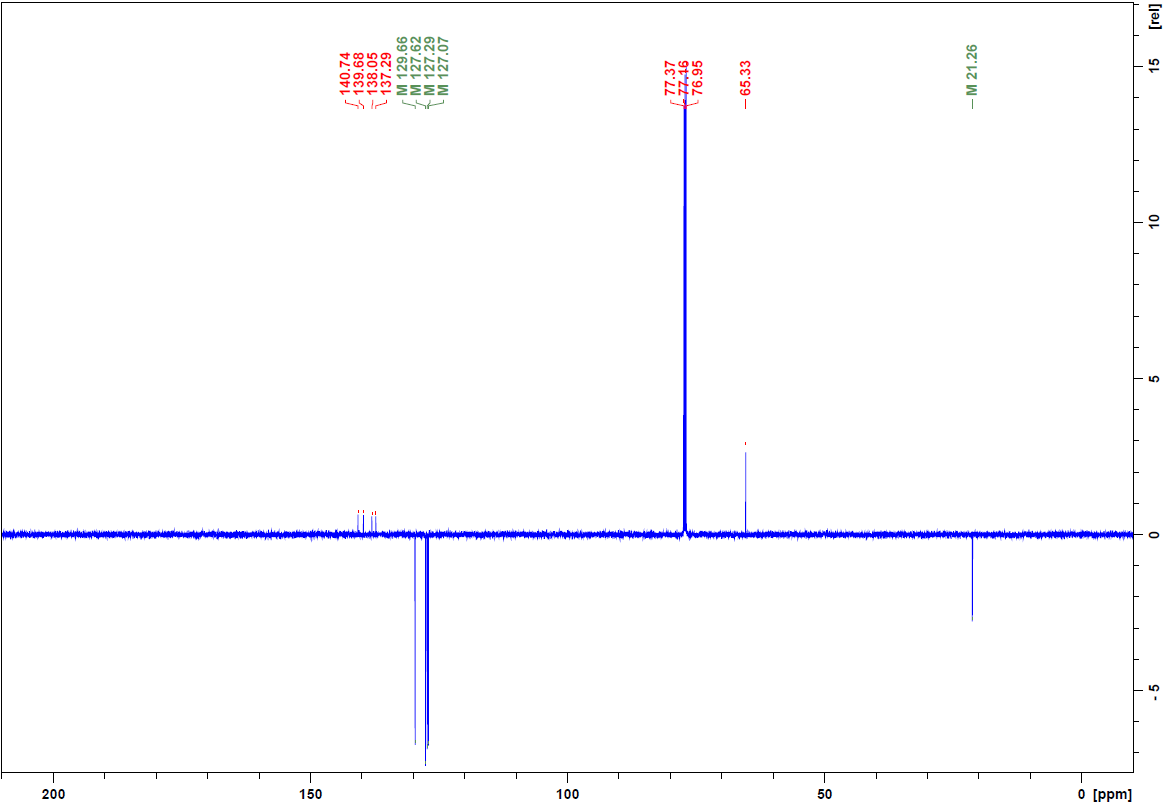


## ^1^H-NMR spectra of compound **XIII**


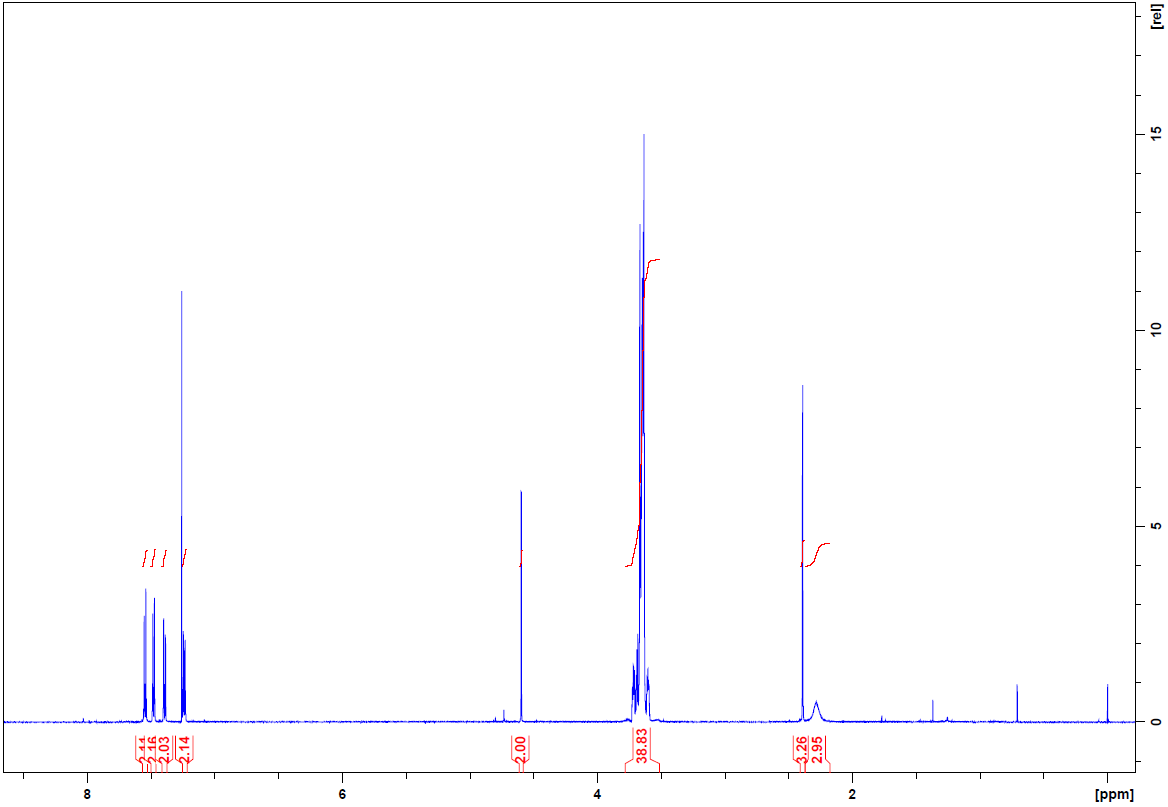

## ^13^C-NMR-Jmod spectra of compound **XIII**


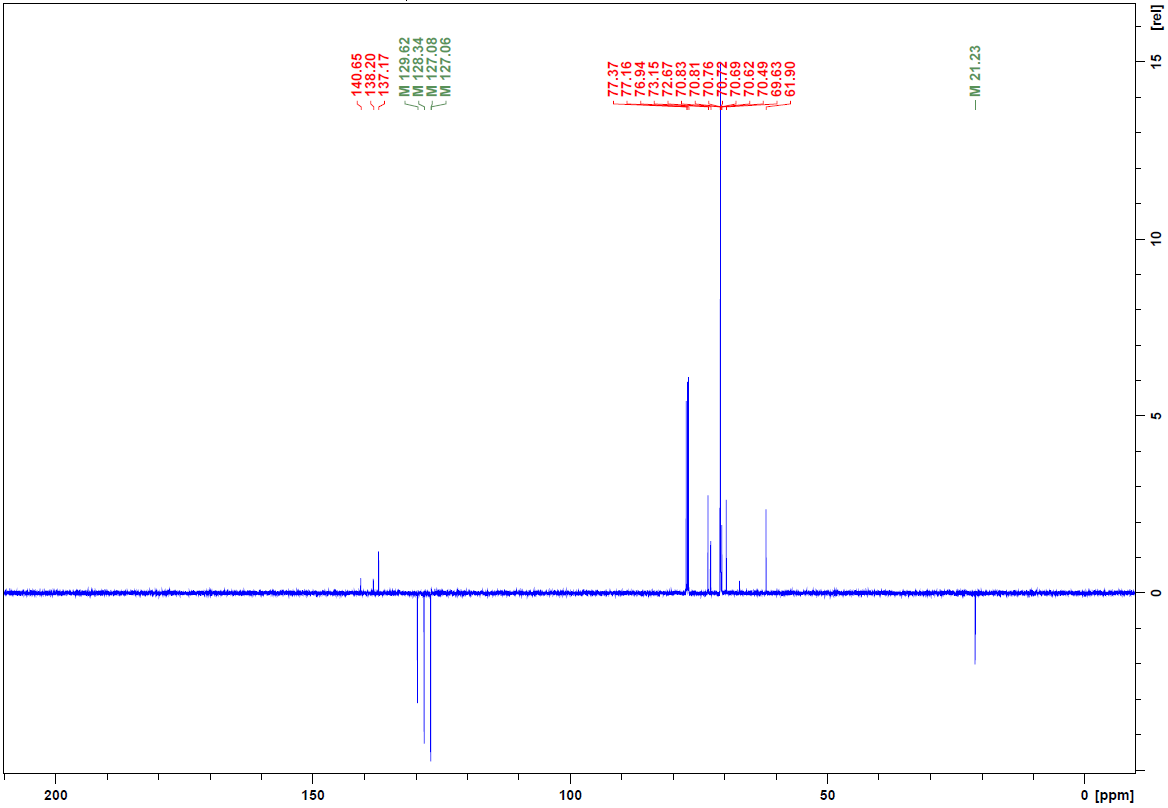


## ^1^H-NMR spectra of compound **XIV**


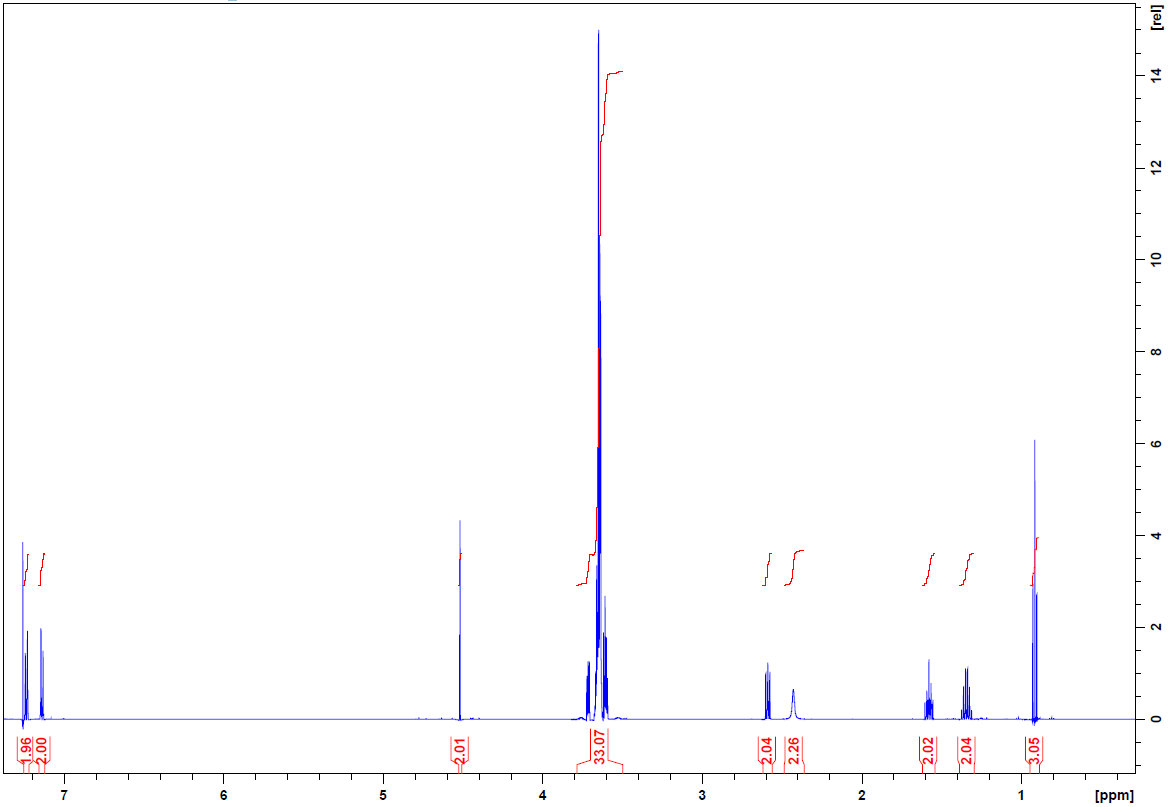

## ^13^C-NMR-Jmod spectra of compound **XIV**


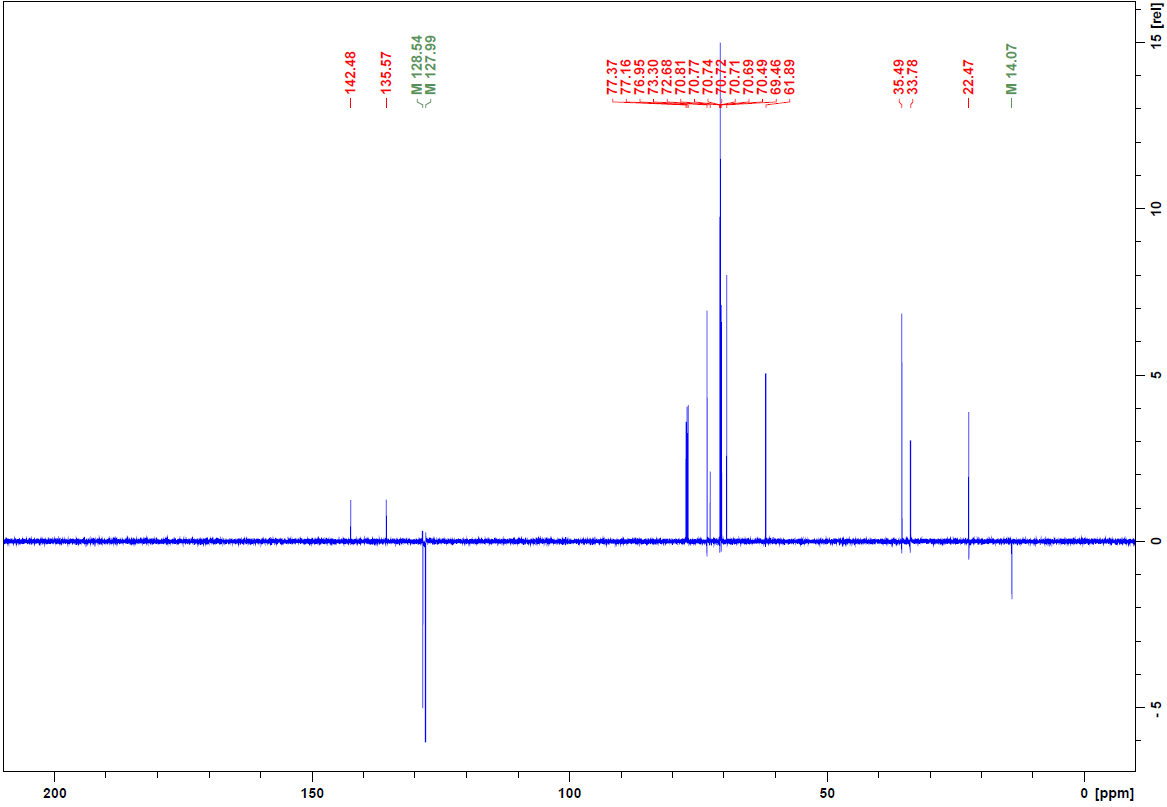


## ^1^H-NMR spectra of compound **XV**


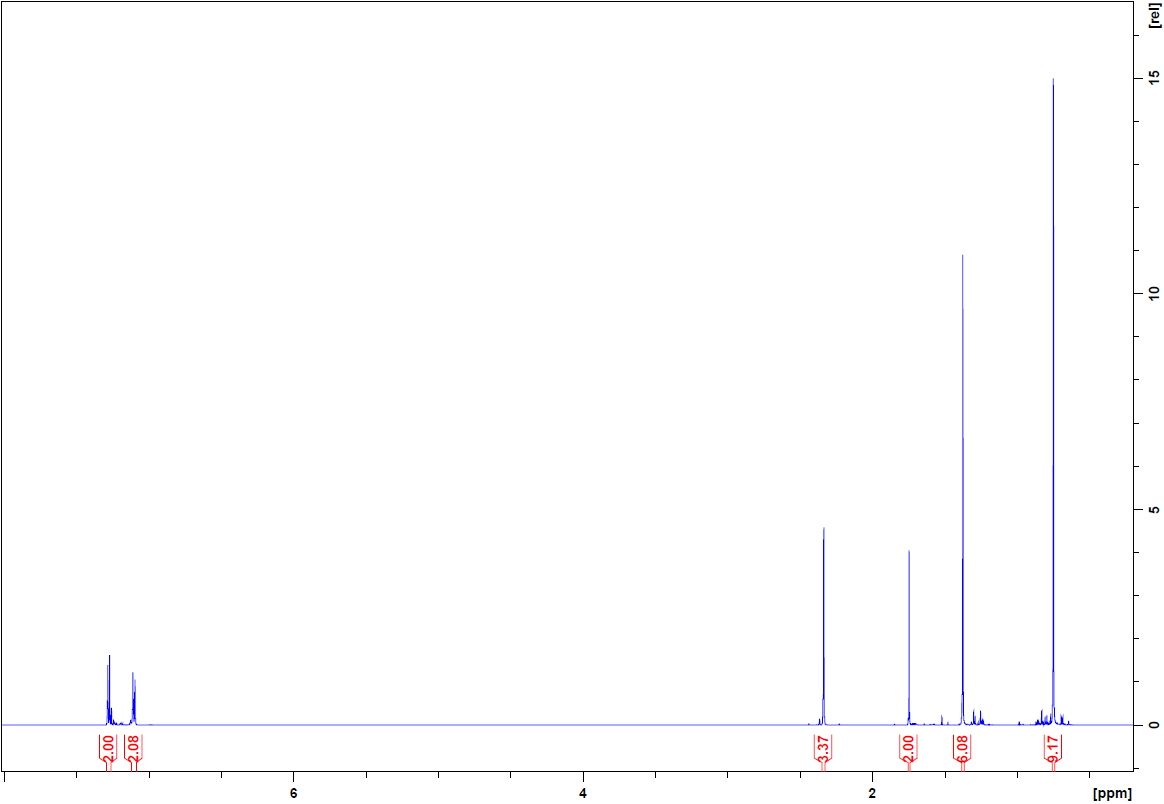

## ^13^C-NMR-Jmod spectra of compound **XV**


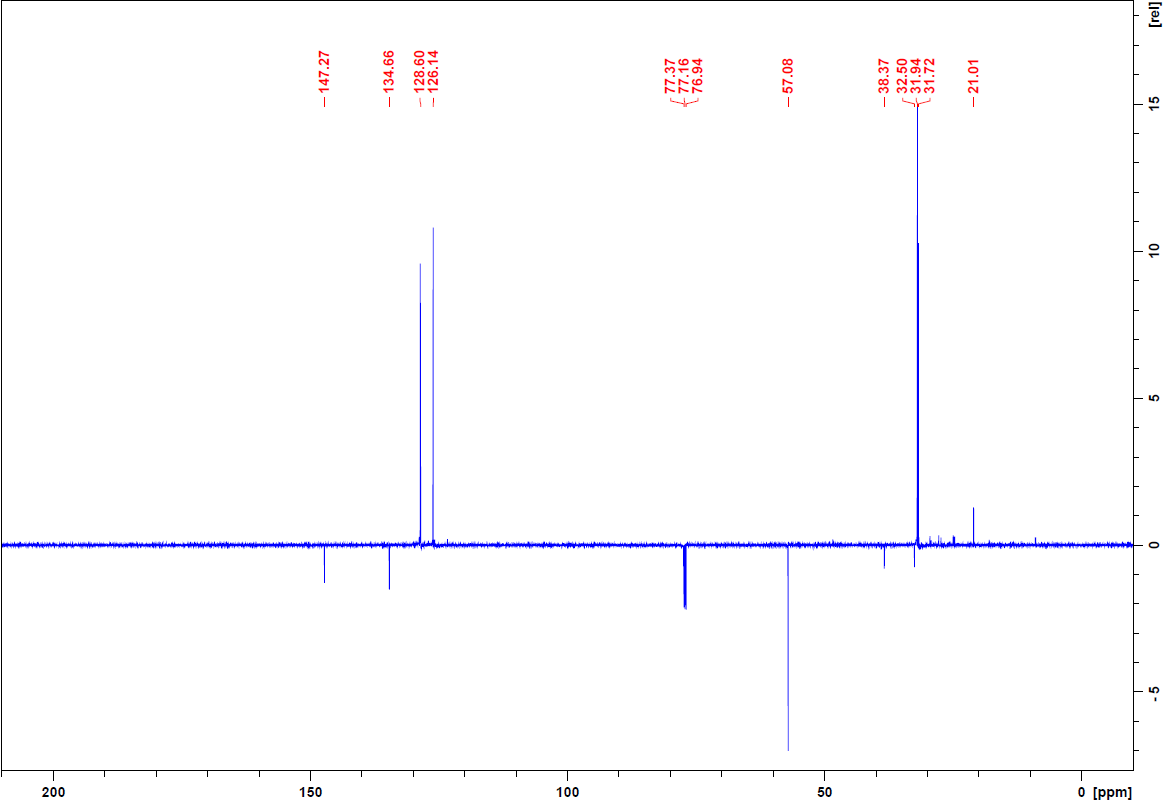


## ^1^H-NMR spectra of compound **XVI**


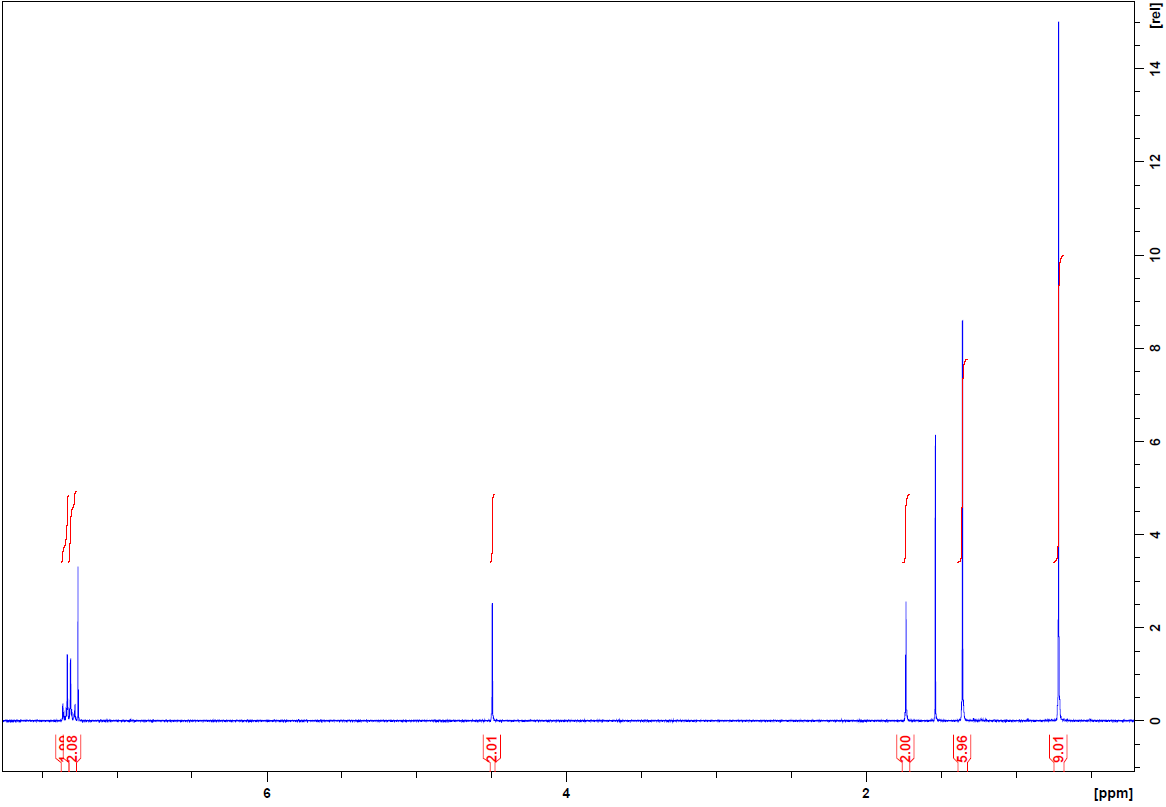

## ^13^C-NMR-Jmod spectra of compound **XVI**


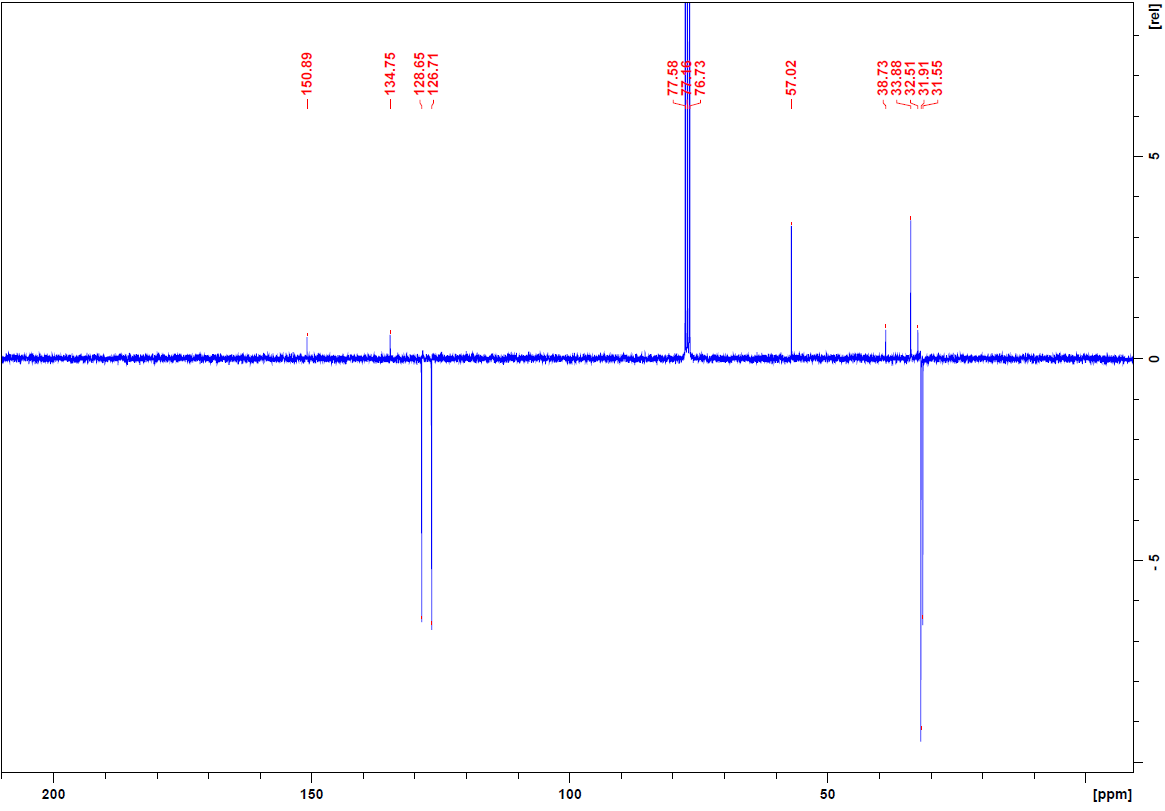


## ^1^H-NMR spectra of compound **XVII**


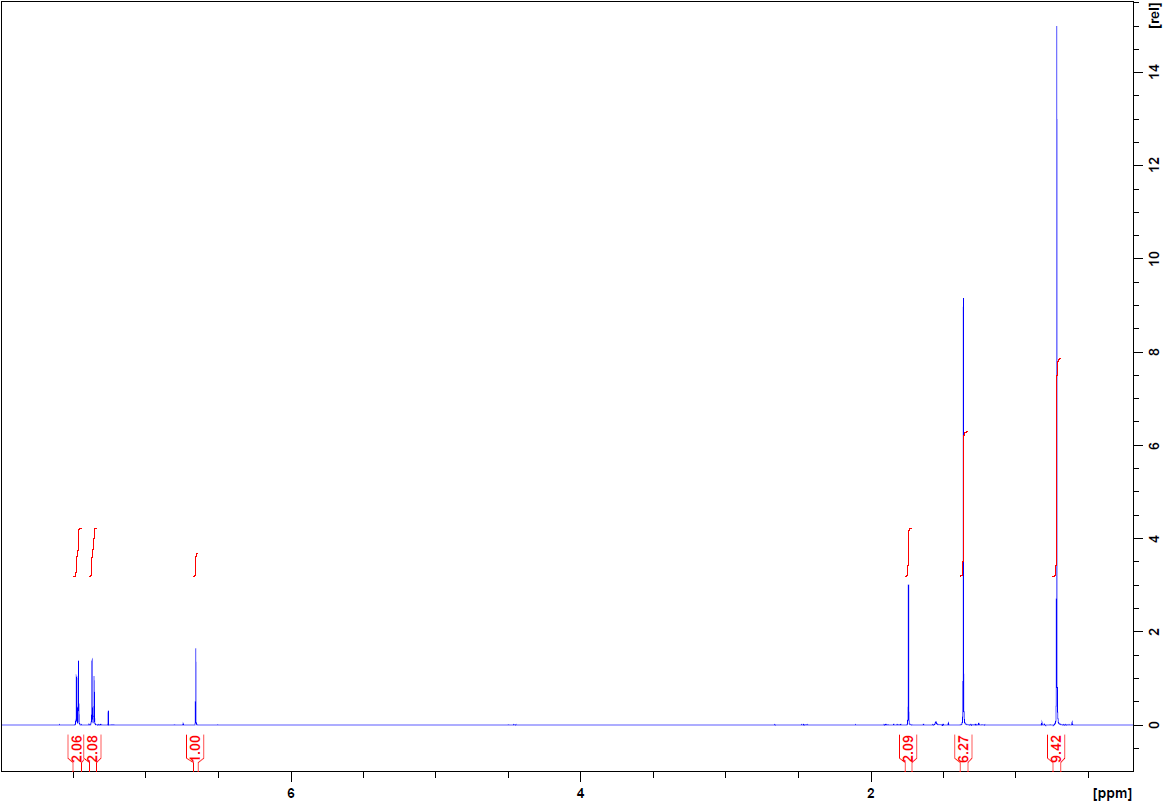

## ^13^C-NMR-Jmod spectra of compound **XVII**


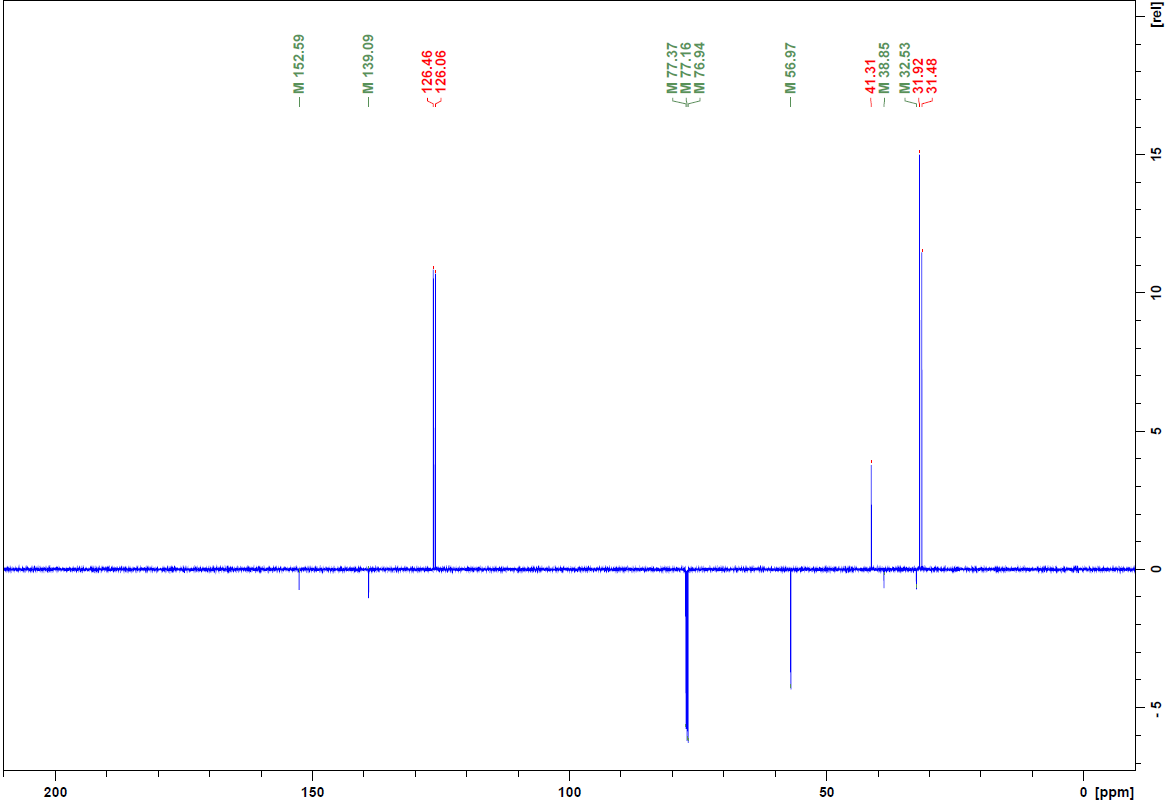


## ^1^H-NMR spectra of compound **XVIII**


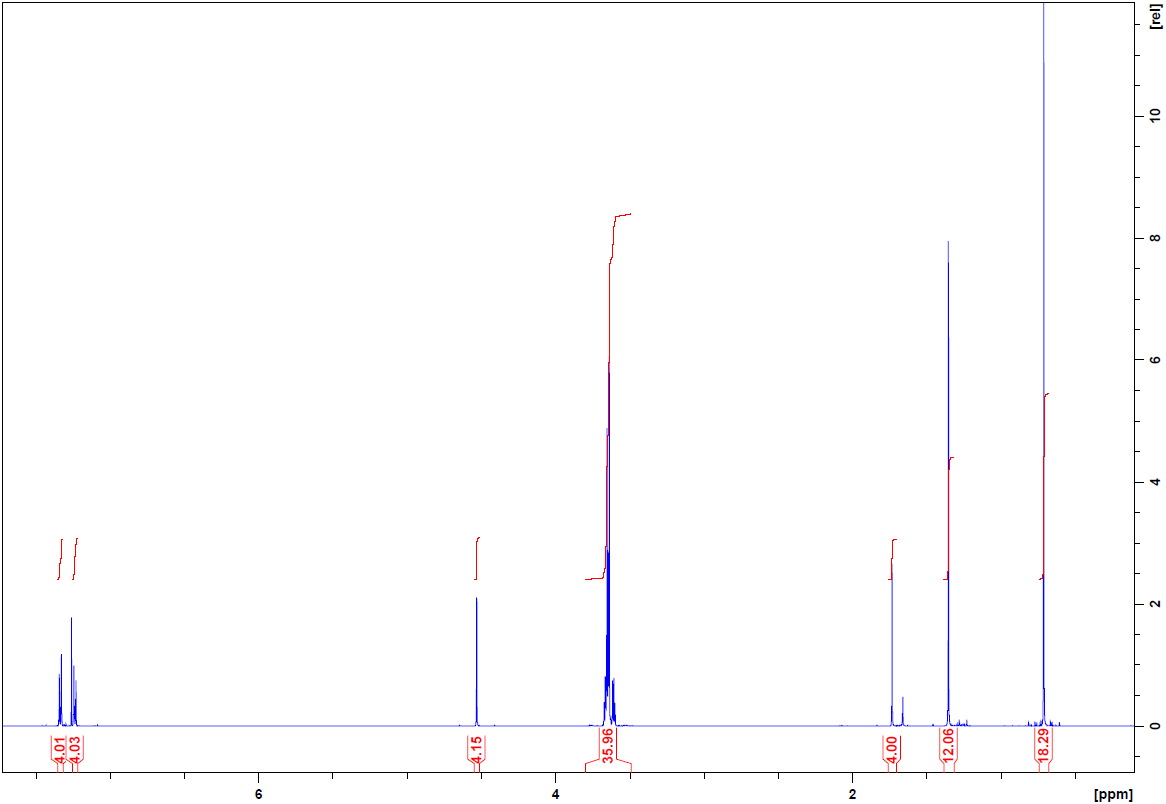

## ^13^C-NMR-Jmod spectra of compound **XVIII**


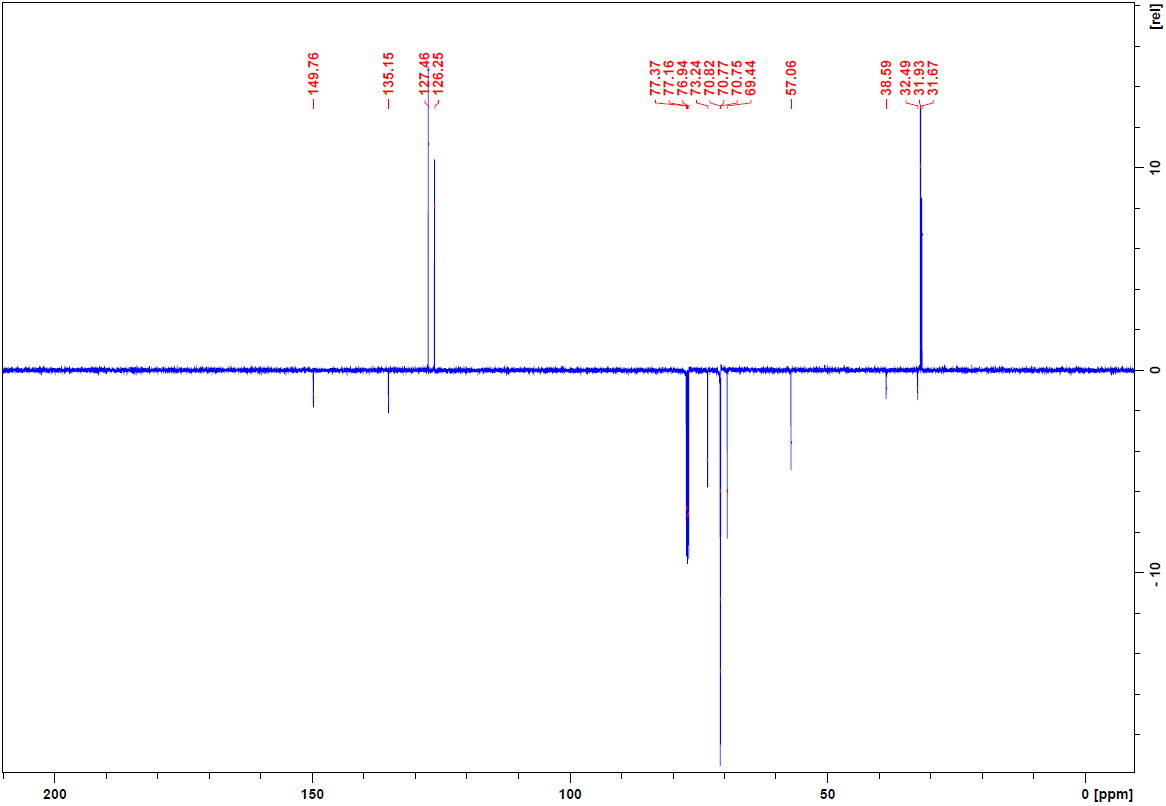

Supplement: Supplementary file 1 — Supplementary information. [file JMV-93-3880-s001.docx]
